# Activated Carbons and Their Evaluation in Electric Double Layer Capacitors

**DOI:** 10.3390/molecules25184255

**Published:** 2020-09-16

**Authors:** Krzysztof Kierzek, Grażyna Gryglewicz

**Affiliations:** Department of Process Engineering and Technology of Polymer and Carbon Materials, Faculty of Chemistry, Wrocław University of Science and Technology, Gdańska 7/9, 50-344 Wrocław, Poland; krzysztof.kierzek@pwr.edu.pl

**Keywords:** supercapacitor, activated carbon, porosity

## Abstract

This review presents a summary of the manufacturing of activated carbons (ACs) as electrode materials for electric double layer capacitors. Commonly used techniques of open and closed porosity determination (gas adsorption, immersion calorimetry, X-ray and neutrons scattering) were briefly described. AC production methods (laboratory and industrial) were detailed presented with the stress on advantages and drawbacks of each ones in the field of electrode materials of supercapacitor. We discussed all general parameters of the activation process and their influence on the production efficiency and the porous structure of ACs. We showed that porosity development of ACs is not the only factor influencing capacity properties. The role of pore size distribution, raw material origin, final carbon structure ordering, particles morphology and purity must be also taken into account. The impact of surface chemistry of AC was considered not only in the context of pseudocapacity but also other important factors, such as inter-particle conductivity, maximal operating voltage window and long-term stability.

## 1. Introduction

The energy stored in electric double-layer capacitors (EDLCs) is related to the electrostatic attraction of ions on the surface of the two electrodes immersed in an electrolyte. Theoretically, the accumulated energy is proportional to the surface area of the electrodes. Activated carbons (ACs) are most often used as the active materials of EDLC electrodes because of their low cost, good electric conductivity, chemical resistivity towards various electrolytes and extremely high specific surface area (SSA). However, the precursor origin, the method of porosity development and the pore size distribution of the resultant ACs also influence the performance of EDLCs.

This review describes several aspects of AC manufacturing and provides general guidelines that facilitate improving the overall process used to obtain the electrode material and achieving optimal capacitance behavior in a given electrolyte. In particular, this information will be helpful in the selection of an organic precursor, the proper method and parameters of activation and the final surface modification. We begin with the classification of organic precursors and the description of the phenomena that occur during their thermal decomposition to better objectivize the impact of the raw material on the porosity development and electrochemical properties of the resultant AC. The methods used to characterize the carbon porosity, including gas adsorption, immersion calorimetry and several spectroscopic techniques, are also briefly discussed. We broadly describe the activation methods commonly used on the industrial and laboratory scales, emphasizing the possible ranges of the yield, general porosity parameters and surface chemistry of ACs. None of the activation methods are enforced, and in all cases, advantages and drawbacks are explicitly presented.

Subsequently, the influence of pore size, carbon structure ordering and particle morphology of the AC on the EDLC performance is discussed in detail based on our own research and other reports. The problem of AC purity is also addressed. We focus on the negative effects of mineral matter and surface functionalities on the long-term performance of EDLCs in both aqueous and non-aqueous electrolytes. The carbon purity is related to the precursor origin and the activation method used.

This review does not presume to offer a complete compendium on the applications of ACs in EDLCs; rather, it summarizes the state of the art and describes the most problematic issues. It should support improved planning of further research on this prospective electric storage system.

## 2. Carbonaceous Materials

### 2.1. Precursors for Activated Carbons

ACs can be prepared with a wide range of porous structures and surface properties depending on the nature of the precursor and the processing method. A large number of raw materials, including wood, coal, peat, lignite and coconut shell, are widely used for the industrial-scale preparation of ACs. In the past decade, intensive studies have been conducted to find low-cost raw materials to decrease the cost of AC manufacturing and, simultaneously, utilize by-products and wastes derived from different industrial areas. Biomass wastes, polymeric wastes, pitch residues and other materials are considered for this purpose [1,2,3]. 

Lignocellulosic materials have been the most widely investigated raw materials for AC preparation. They consist mainly of cellulose, lignin and hemicellulose, with other components, such as simple sugars, proteins, starches and lipids, constituting a large minority. The biopolymer composition of lignocellulosic materials varies greatly depending on their nature, with major components exhibiting the following ranges: 30–50 wt% cellulose, 20–35 wt% lignin and 20–30 wt% hemicellulose (Table 1). The composition of the raw material affects the product yield, which is related to the different reactivities/thermal stabilities of biopolymers during carbonization. For example, for lignin, the yield of carbonization is in the range of 40–45 wt%, whereas it only 20–30 wt% for cellulose, depending on the heat treatment temperature (HTT). Therefore, from the product yield perspective, a lignin-rich raw material is most suitable for the production of AC.

The utilization of low-cost biomass can reduce the cost of AC production; however, a stable source supply in terms of physicochemical properties and large quantities are required to ensure economically attractive AC production on an industrial scale. Some precursors used are interesting because of their specific composition. For example, chitosan has a nitrogen content exceeding 8 wt% [12]. As a result, the ACs produced from these precursors are distinguished by high contents of nitrogen functional groups in their structures, which can be favorable for pseudocapacitors. 

Polymeric wastes, such as poly(ethylene terephthalate) (PET) [13,14,15], polyacrylonitrile (PAN) [16,17], poly(furfuryl alcohol) (PFA) [3], polystyrene [18], phenol-formaldehyde resin [19], styrene divinylbenzene resin [20], melamine–formaldehyde resin [21] were used for AC preparation. However, some of these wastes (e.g., PET [22]) exhibit relatively low carbonization yields, which is a serious drawback of their use for this purpose. Regardless of the method of AC preparation, the char yield is a relevant factor in determining the economic attractiveness of the precursor. The carbonization yields for various raw materials are presented in Table 2. Char yields obtained on carbonization of various lignocellulosic, polymeric and carbonaceous raw materials. It is worth noting that the pre-oxidation of some polymers can be a very effective way to increase the solid residue yield [23,24].

Another precursor feature that is very important for AC production is related to the presence of mineral substances. A low mineral matter content in the precursor is particularly desirable if the AC is produced through physical activation because inorganic matter continuously accumulates during carbonization and subsequent carbon gasification. Therefore, for the production of ACs from coal, the ash content in the raw material should not exceed 5–7 wt%, and thus, a special selection or pretreatment of coal is required. This issue can be easily overcome by using wood and fruit shells because of their naturally low ash contents (<1 and <4 wt%, respectively).

In summary, ACs can be prepared from virtually any carbonaceous precursor. However, the most suitable features for AC precursors are a polymeric or macromolecular structure, high carbon content, low inorganic matter content and high carbonization yield.

### 2.2. The Structure of Non-Graphitic Carbons

**Figure 1:**
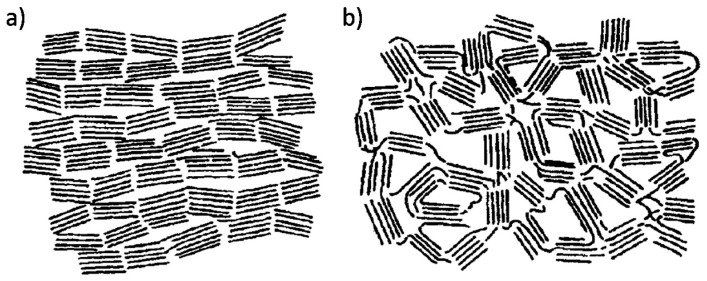
Schematic models for the microstructure of *graphitizable* carbons (**a**) and non-*graphitizable* carbons (**b**). Reproduced from [25] with permission of The Royal Society (UK) © 1951.

Non-graphitic carbons can be divided into two categories—non-graphitizable and graphitizable carbons—depending on their ability to develop a three-dimensional graphite crystal structure during HTT. The differences in their behavior are attributable to their different microstructures. This classification of carbon materials was proposed by Franklin [25] based on an X-ray diffraction study of polymer chars. Schematic models of graphitizable carbon (a) and non-graphitizable carbon (b) are shown in Figure 1.

The structure of graphitizable carbons consists of graphene crystallites which are arranged in a near-parallel mode. The heat treatment of graphitizable carbons increases the structural ordering by eliminating heteroatoms and other structural defects to form a three-dimensional graphitic structure. In contrast, carbon materials that cannot form a graphitic structure under high-temperature treatment up to 3000 °C in inert conditions are referred to as non-graphitizable carbons. The structure of non-graphitizable carbons consists of randomly oriented graphene crystallites that are cross-linked by disordered carbon, creating open pore space.

For the development of graphitizable carbons, the formation of a liquid crystal phase called the carbonaceous mesophase during a fluid stage in carbonization is required [26]. This mesophase is formed during the carbonization of coal-tar pitch, petroleum pitch, coking coal and certain polymers, such as polyvinylchloride. Non-graphitizable carbons are obtained from natural or synthetic organic materials, such as various lignocellulosic materials, non-fusing coals and thermosetting polymers, that do not pass through a fluid stage during carbonization. As a result, the macromolecular or polymeric structures of these materials are retained after heat treatment, resulting in porous structures.

**Figure 2:**
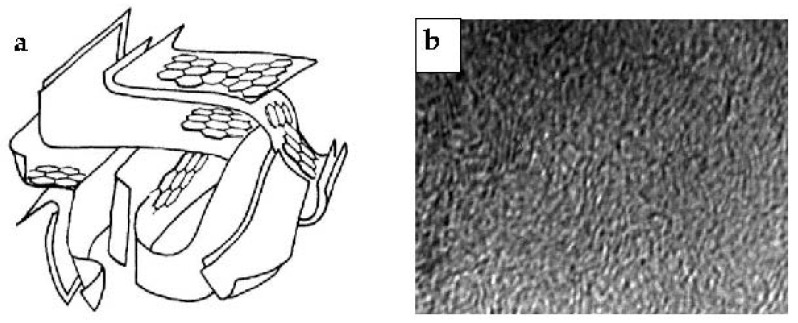
Schematic representation of the structure (**a**). Reproduced from [27] with permission from Elsevier © 1990, and a typical HRTEM image of an AC (**b**).

ACs are non-graphitizable carbons. An AC model elaborated based on high-resolution transmission electron microscopy (HRTEM) was proposed by Stoeckli (Figure 2a) [27]. This model describes the three-dimensional network as a loose packing of curved aromatic sheets bonded together with many structural defects. The structure of AC resembles wood shavings, and the resulting spaces, which are often slit-shaped, correspond to micropores. Mesopores and macropores result from the voids between aggregates. HRTEM lattice-fringe image of a commercial AC is shown in Figure 2b.

ACs consist of carbon, hydrogen, oxygen, nitrogen and, if it is present in the precursor, sulfur. The carbon and oxygen contents vary in the ranges of 70–95 wt% and 5–25 wt%, respectively, and are mainly dependent on the activation method used. Heteroatoms occur in organic functional groups located at the edges of the basal planes. Oxygen-containing groups are mostly responsible for the carbon surface properties because of their abundance in the AC. Their presence enhances the wettability of the carbon surface, which is of particular importance for AC-based electrodes in supercapacitors operating in aqueous solutions. In addition to the organic matter, mineral matter is present in ACs but as a significant minority and is usually expressed as the ash content. The presence of mineral matter is strongly undesirable in ACs used as electrode materials for supercapacitors. This issue will be discussed in Section 4.4. 

### 2.3. The Porous Texture in Carbon Materials

**Figure 3:**
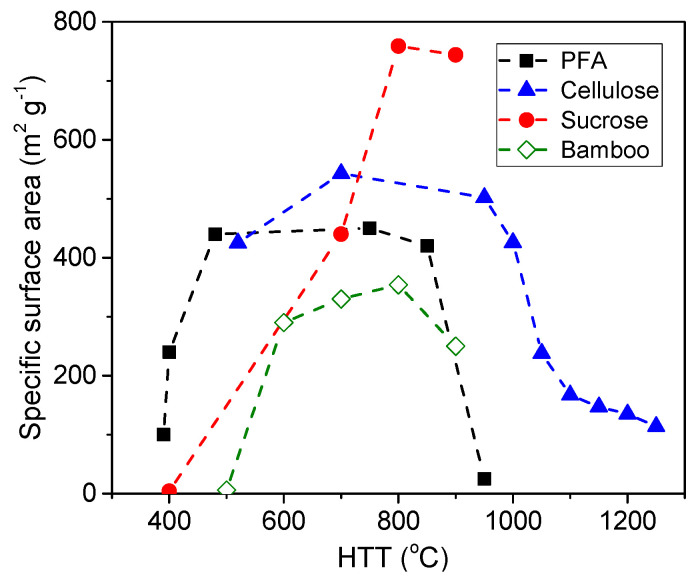
The variation of specific surface area with HTT for the carbonization of polyfurfuryl alcohol (PFA, reproduced from [28] with permission from Elsevier, © 1964), sucrose (reproduced from [29] with permission from Elsevier © 2014), bamboo (reproduced from [30] with permission from Elsevier © 2014) and cellulose.

The thermal decomposition of organic materials causes the transformation of a polymeric or macromolecular system into a three-dimensional network of carbon atoms. The result is not a continuous phase but one that is rich in void spaces. The pristine porous texture generated is strongly dependent on the nature of the raw material and the carbonization temperature. Generally, the carbonization of a macromolecular precursor, such as coal, leads to low-porosity carbons with a pore width in the range of ultramicropores (<0.7 nm). In contrast, the chars from polymeric organic materials, such as biopolymers and synthetic polymers, have SSAs of hundreds of square meters per gram and exhibit a strong dependence on the final HTT. The porosity usually increases very rapidly as the temperature increases to 700–800 °C. Above this value, a sudden decrease in the porosity is observed, which is related to a significant hydrogen release and carbon structure shrinkage. This phenomenon is illustrated in Figure 3.

The porosity derived from simple carbonization is usually too low for the reasonable application of the resulting char and must be further developed by an activation process, as described in Section 3. Regardless of the origin of the carbon porosity, its precise determination is very important. Direct analysis of the pore structure at the sub-nanometer scale is difficult and requires expensive equipment, such as HRTEM, in combination with the application of a complex numerical algorithm to a series of lattice-fringe images [31]. The physical adsorption of gases is the most frequently proposed technique in the literature. In this method, the experimental isotherms of selected gas molecules, such as N_2_ at 77 K and CO_2_ at 273 K (or 298 K), are analyzed. The nitrogen adsorption allows determining the pore size in the range of 0.7–50 nm, including wider micropores (<2 nm) and all mesopores (2–50 nm). CO_2_ molecules facilitate probing pores that are smaller than 0.4 nm. The upper limit of the CO_2_ adsorption analysis remains debatable. An upper limit of 1–1.5 nm is most frequently suggested, depending on the temperature and adsorption model used. Generally, CO_2_ adsorption covers only the ranges of ultramicropores (<0.7 nm) and narrow micropores and is complementary to the N_2_ adsorption analysis of carbons [32]. The raw isotherm data can be interpreted by a series of adsorption models to calculate the porosity parameters. The most often reported parameters are as follows:

- S_BET_—the Brunauer-Emmett-Teller (BET) specific surface calculated from N_2_ adsorption isotherm data based on the BET model. BET theory has been verified using many mesoporous materials. However, recently, it has been strongly criticized when applied to the analysis of microporous carbons because it can significantly over-estimate the real surface area [33]. Despite this drawback, in the literature, this model is commonly used to differentiate between ACs in a series.
- S_DFT_—the SSA calculated from density functional theory (DFT) based on N_2_ and CO_2_ adsorption isotherm data. This parameter seems to be more reliable than S_BET_, and many reports have demonstrated the convergence of S_DFT_ and the real surface areas of materials [34,35]. The DFT method is also commonly used to determine the pore size distribution precisely. Two implementations of DFT are described in the literature: Non-Local DFT (NLDFT) [36] and Quenched Solid DFT (QSDFT) [34]. These two analyses give slightly different values and are selected arbitrarily.
- V_0_ and S_0_ (or V_DR_ and S_DR_)—the volume and surface area of micropores based on the Dubinin-Radushkevich (DR) model. These values can be determined from N_2_ and CO_2_ adsorption isotherm data. The volume of the micropores is directly calculated from the DR equation. In contrast, the surface is the derivative parameter estimated from V_0_ based on the assumption of a particular pore shape (slit or cylindrical).
- L_0_—the mean micropore size derived from the V_DR_ based on the empirical equation proposed by Stoeckli [37]. For microporous carbons with a mean pore size smaller than 1.8 nm, the equation is as follows:
  (1) $$L_{0}=\frac{10.8}{E_{0}-11.4}$$
  For carbon with a wider pore size, the mean pore size is calculated according to the following equation:
  (2) $$L_{0}=\frac{13.7}{E_{0}-9.7}$$
  where E_0_ is the characteristic energy of adsorption.
- V_T_—the total pore volume calculated from N_2_ adsorption data. This parameter is directly related to the volume of liquefied nitrogen stored in the pores, assuming ideal capillary condensation. It is calculated for the last adsorption point of the isotherm or at a partial pressure of approximately 0.96 p p_0_^−1^, which corresponds to a pore size of 50 nm.

Immersion calorimetry can be used to characterize the carbon porosity as an alternative to gas adsorption isotherms. This technique is based on measuring the heat of wetting the carbon with a solvent or dilute solutions of various compounds, such as methanol, benzene or an aqueous solution of phenol. This approach assumes the formation of a physically adsorbed monolayer of molecules. Then, the heat (enthalpy of immersion) is directly related to the surface area of the carbon accessible to the wetting molecules [38,39]. The total (internal and external) surface area is determined in this method, but the development of microporosity can be calculated using the Dubinin model [40]. The probe molecules should be selected carefully to fit the size of the pores and avoid chemisorption because the excessive heat related to chemisorption can distort the data.

It must be noted that gas adsorption and immersion calorimetry techniques can only be used to analyze the *open porosity*, i.e., the system of pores with entries on the outer surface of the carbon particle. Moreover, the width of the entries must be greater than the size of the probe molecule. These techniques are useless for the most highly disordered chars (before activation) derived from non-graphitizable precursors, such as lignin-cellulose materials and synthetic resins, for which very small void spaces in a large volume are deeply embedded inside the carbon structure. This texture is called *closed porosity* [41]. The closed spaces have a minor contribution to the porosity of AC and are often ignored because they do not play a role in most material applications, such as pollutant adsorption and EDLCs. However, the closed porosity can lower the packing density of the carbon. Additionally, under some conditions, the pores can be filled with small metallic atoms, such as Li and Na. Accordingly, the closed porosity is beneficial for the application of carbons as anodes of Li-ion or Na-ion cells [42]. Furthermore, such porous systems can respond to small-angle X-ray scattering (SAXS) [43,44,45] and small-angle neutron scattering (SANS) [43,46,47], in which the probes are X-ray photons and neutrons, respectively. These techniques measure the heterogeneity of the carbon structure with scattering dimensions in the range of 1–200 nm and a resolution of 0.2 nm. The direct determination of closed pores by HRTEM imaging is also possible.

## 3. Activation Process

**Figure 4:**
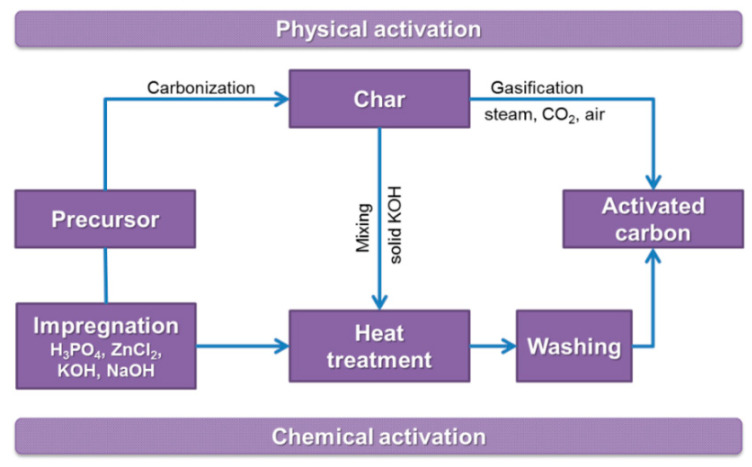
AC preparation by physical and chemical activation.

As mentioned previously, the chars produced from organic precursors by pyrolysis usually show poor pristine porosity that is mainly closed. Therefore, the SSAs of these chars are too low and the pores too narrow for any reasonable application. The process through which the existing porosity is developed or a new porous system generated is called “activation”. Several industrial or laboratory activation processes are used for the development of porosity in carbon. Generally, these processes can be divided into two groups—physical (thermal) activation and chemical activation—that differ in their pore evolution mechanism. A given process cannot be uncritically applied to any precursor. Moreover, the final products show varied pore size distributions and surface chemistries. The possible activation routes are schematically presented in Figure 4.

### 3.1. Physical Activation

Physical activation is commonly used on an industrial scale. In this process, the char is selectively gasified by carbon dioxide or water vapor (steam). This process is conducted mainly at 800–1000 °C, and carbon monoxide and hydrogen are generated as side products. The overall reactions of carbon with CO_2_ and steam to remove carbon atoms from the network within solid carbon are as follows:

C + CO_2_ ⇔ 2CO ΔH = 132 kJ mol^−1^

C + H_2_O ⇔ H_2_ + CO ΔH = 159 kJ mol^−1^

These reactions are endothermic, and therefore, extra heating is necessary to maintain the process temperature. Another solution is to inject a controlled amount of air into the reactor to partially burn off the carbon and gases produced during activation. This injection can enhance the process effectiveness since the carbon precursor is usually cheaper than the natural gas used for the external heating of the reactor. In this case, a combined process of CO_2_ and steam activation can occur. 

Three main factors influence the rate of the gasification process and, consequently, the properties and porosity of the resulting carbon: the gasification temperature, the pre-carbonization temperature of the organic precursor and its origin.

#### 3.1.1. Effect of the Gasification Temperature

The gasification process is based on the heterogeneous reaction between gas molecules (CO_2_ and H_2_O) and solid carbon. The process kinetics is controlled by the rate of the chemical reaction and diffusion. At a lower temperature, the reaction rate is determinative. The oxidative molecules have sufficient time to penetrate the carbon structure before gasification, and as a result, a more homogenous product with a narrower pore size distribution can be produced. At a higher temperature, the process is mostly controlled by the diffusion rate of the reactants to the carbon surface. Gas molecules react preferentially with carbon at the outer particle surface, leading to external particle burning, resulting in low process yields and ACs with unusable porosity textures. Steam activation should be conducted at temperatures lower than 1000 °C to maintain the gasification process under chemical control. In practice, the temperature of the process should be optimized to obtain a product with the desired porosity after a reasonable treatment time.

#### 3.1.2. Effect of the Pre-Carbonization Temperature

A two-stage activation process is applied in conventional industrial manufacturing. Before gasification by CO_2_ or steam, the organic precursor is gradually heat-treated (carbonized) in an inert atmosphere to remove most of the volatile matter. Then, the resultant char is allowed to contact the activating gas. The reactivity of the char depends on the final carbonization temperature, which is directly linked to the carbon structure ordering. At this stage, the typical temperature ranges between 600 and 900 °C. Generally, a higher carbonization temperature decreases the rate of gasification and allows a product with limited porosity development but a narrower pore size distribution to be obtained.

#### 3.1.3. Effect of the Precursor Origin

The precursor origin is also related to the reactivity of the char produced in the carbonization stage. As described in Section 2.2, the chemical nature of the organic material determines its ability to generate well-ordered carbon during thermal decomposition. Polymeric precursors, such as lignocellulosic materials and synthetic polymers, yield chars with more structural defects than macromolecular materials. Moreover, significant pristine porosity is created during the carbonization of polymeric precursors. Carbons with such defect-filled structures are characterized by much higher gasification rates than well-ordered carbons during activation. 

Various natural and synthetic organic materials can be used as raw materials in the physical activation process. In large-scale production, coal, wood or shells are commonly used because of their relatively low price and high availability. If an AC with low mineral matter content is needed, a cellulose-derived precursor is usually chosen because doing so allows omitting the cost-effective demineralization process. This type of material is often selected when a granular product of high mechanical strength is required. At the laboratory scale, the list of materials activated with steam or CO_2_ seems unlimited. Despite the low process rate and limited porosity development, even well-ordered precursors, such as cokes from coal-tar pitch, are subjected to physical activation to obtain specific electric properties derived from the precursor [48,49]. 

#### 3.1.4. Carbon Dioxide Versus Steam

**Figure 5:**
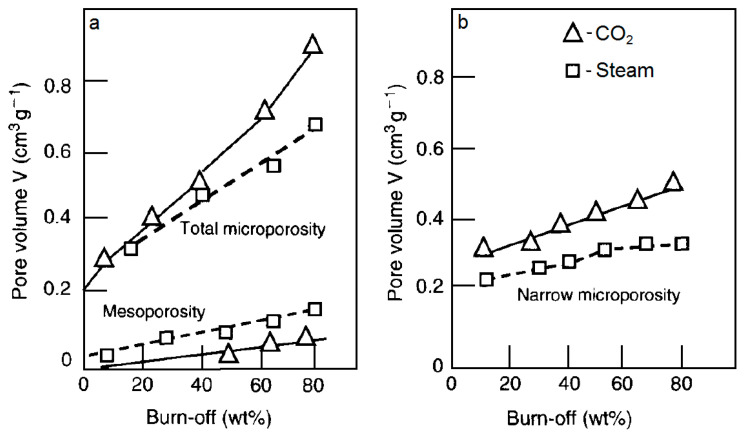
Evolution of micropore and mesopore volumes (**a**) and volume of narrow micropores (**b**) as a function of burn-off in CO_2_ and steam from olive stone. Reproduced from [50] with permission from Elsevier © 1995.

Since the role of the process temperature and the nature of the precursor are controlling factors, a detailed comparison of the porosity developed by carbon dioxide and steam activation under otherwise fixed experimental conditions is required. Generally, precursors show higher reactivity towards steam than carbon dioxide. However, at the same process rate achieved by temperature optimization, carbon dioxide facilitates obtaining ACs with higher and narrower porosities, as demonstrated by Rodriguez-Reinoso et al. [50]. For both activation agents, a continuous increase in the pore volume with char burn-off is observed. The development of microporosity is favored in both cases, but CO_2_ allows producing carbons of higher porosity. Moreover, the higher contribution of ultramicropores (determined from the CO_2_ adsorption isotherm) and the lower amount of mesopores are relevant in CO_2_ gasification (Figure 5).

**Figure 6:**
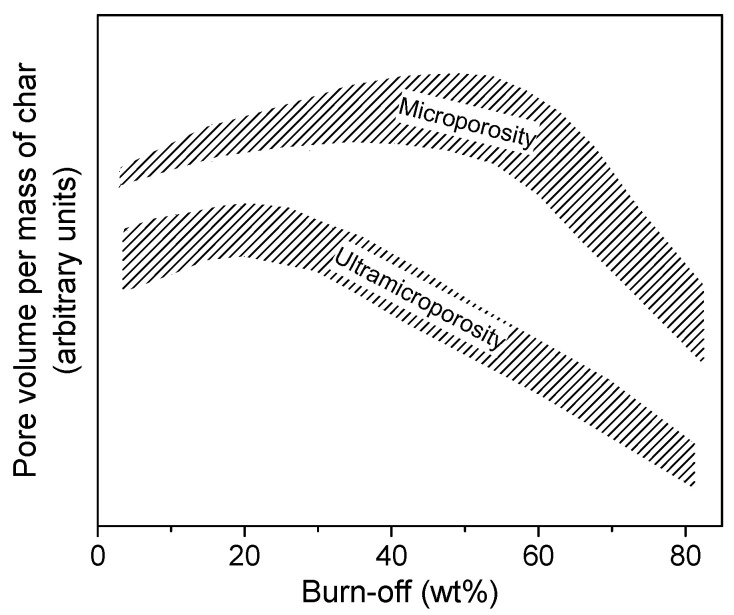
Typical variation of microporosity (<2 nm) and ultramicroporosity (<0.7 nm), expressed per unit weight of char, as a function of burn-off in physical activation.

Considering process efficiency, it is more convenient to analyze the porosity of the product in terms of the unit weight of the starting char rather than the weight of the resultant AC. A general tendency is schematically plotted in Figure 6. Narrower pores are created with the highest yield at the beginning of the gasification process. However, the optimal total microporosity development of the char occurs at approximately 50% burn-off.

The porosity development is the major feature of ACs. Nevertheless, in some applications, such as supercapacitors, the surface chemistry of the material is also important. A typical physical activated AC contains only 3–5 wt% oxygen (excluding moisture). Generally, the amount of oxygen groups retained on the carbon surface is inversely proportional to the gasification temperature. However, compared to steam activation, CO_2_ activation at a similar temperature results in a slightly higher number of oxygen functional moieties with better thermal stability [51]. 

In summary, physical activation is a cheap and commonly used process of manufacturing microporous ACs for general-purpose applications, such as water and air treatment. However, this process has significant limitations. It is difficult to select a precursor and tailor the process parameters to obtain carbon with a SSA above 2000 m^2^ g^−1^, a narrow pore size distribution and a reasonable yield. Chemical activation seems to be a much better option to fulfill these objectives.

### 3.2. Chemical Activation

Briefly, “chemical activation” is the method of porosity development that involves the co-carbonization of a precursor with a solid or liquid chemical. The chemical agent does not selectively remove carbon atoms as in the case of thermal activation but rather interacts with the precursor according to mechanisms depending on the activation agent. The most common chemical agents used are phosphoric acid, zinc chloride and potassium hydroxide. The first two act as dehydrating agents, whereas KOH acts as an oxidant. 

#### 3.2.1. Activation with Alkali Metal Hydroxides

In practice, KOH and K_2_CO_3_ are commonly used as starting reagents in this process because of their high reactivity towards a wide range of precursors. Sodium hydroxide and sodium carbonate are more efficient for the activation of materials with low structural order [52,53,54]. Activation with KOH begins at temperatures between 300 and 400 °C depending on the precursor, but a reasonable process rate is observed above 600 °C, after the organic precursor has been transformed into char. This process is based on three phenomena. First, the less-stable carbon groups are dehydrogenated and eliminated. Next, the carbon structure undergoes selective oxidation to CO and CO_2_. The reaction of carbon with the evolved CO_2_ proceeds at temperatures above 800 °C, resulting in pristine porosity [55]. Simultaneously, KOH and K_2_CO_3_ are reduced to metallic potassium, which intercalates between the graphene layers. Further heating drives the metal out of the material, generating slit-shaped porosity. For some precursors, this de-intercalation process is so vigorous that the pristine porosity structure created by oxidation is destroyed, and the char particles disintegrate into low-porosity fine powders. The whole process is complex but can be summarized by several reactions:

2 = CH_2_ +2KOH = 2K + 2CO + 3H_2_ (R1)

4KOH + C = 4K + CO_2_ + 2H_2_O (R2)

4KOH + 2CO_2_ = 2K_2_CO_3_ + 2H_2_O (R3)

K_2_CO_3_ + 2C = 2K + 3CO (R4)

Potassium carbonate is formed as a by-product during CO_2_ capture (R3), and thus, even when pure KOH is applied as the starting activation agent, the real process is based on the concurrent reactions of carbon with KOH and K_2_CO_3_. Gaseous hydrogen can also be generated, especially when a macromolecular precursor with a high H/C content is subjected to activation.

It is relatively easy to obtain microporous ACs with very high BET surface areas, even above 3000 m^2^ g^−1^; narrow pore size distributions; and low burn-off of the char [56]. However, the necessity of separating carbon from the solid side products is the main drawback of this process. The safe utilization of hydrogen and metallic potassium vapor and the cost of KOH are also relevant issues. These factors determine the low overall economy of the KOH activation process. Actually, KOH activation is not applied on the industrial scale.

At the laboratory scale, the KOH activation process is easy to perform. This process is very effective and commonly used for the activation of various precursors, such as lignocellulosic materials, synthetic polymers, pitch-derived coke and other materials. Several factors influence the porosity of the resulting ACs. The most important factors are the reaction temperature, the KOH: precursor mass ratio, the nature of the precursor, the reagent mixing method, the soaking time and the inert gas flow rate.

##### The Impact of the Reaction Temperature

The activation temperature has the strongest impact on the porosity development of ACs. For precursors with low carbon contents, such as lignite and lignocellulosic materials, their dehydrogenation by KOH starts at 300 °C (reaction R1). In contrast, precursors with lower H/C ratios, such as pre-carbonized coal and anthracite, react with KOH at temperatures above 400–450 °C. In all cases, the dehydrogenation reaction rate peaks at 600 °C [57]. This process is linked to the creation of very high porosity. An example is given in Table 3. When a physical mixture of bituminous coal and KOH at a mass ratio of 1:4 is carbonized at 520 °C for 1 h, microporous AC with a BET surface area of over 1700 m^2^ g^−1^ is obtained. In contrast, coal carbonized without KOH under the same thermal conditions gives a non-porous product. Interestingly, in both processes, the mass loss of the precursor is similar (approximately 23 wt%).

**Figure 7:**
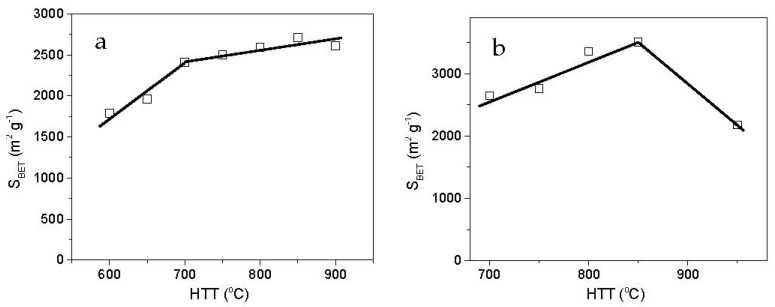
Effect of reaction temperature on the BET surface area of coal-tar mesophase (**a**) and of anthracite (**b**) activated with KOH at a precursor/KOH ratio of 1:3 for 1 h (**a**) and 2 h (**b**).

Further increasing the reaction temperature allows obtaining carbons with higher SSAs, but the relationship is not linear. For precursors with low hydrogen contents, such as high-temperature cokes, the highest porosity development is usually attained at 700–800 °C [55]. The oxidative gasification of the carbon structure and potassium intercalation (reactions R2–R4) are then favored. The variation of porosity development during coal-tar mesophase activation by KOH in the temperature range of 600–900 °C is shown in Figure 7a and Table 4. 

Raising the temperature above 700 °C seems to be less effective. Only a slight increase in the BET surface area is observed since micropore widening and noticeably higher burn-off occur. A reasonable reduction in the porosity development is observed at 900 °C. A similar tendency was reported by Lozano-Castello et al. [58] during the KOH activation of anthracite at 700–950 °C. For the resultant ACs, the BET surface area and mean micropore size are linearly related to the HTT in the temperature range of 700–850 °C (Figure 7b). Since anthracite is more reactive toward KOH than the mesophase, HTT exerts a stronger effect on the porosity development and pore widening.

In the case of anthracite, a decrease in the carbon porosity and an increase in the mesopore contribution also occur above 850 °C because of additional carbon gasification by the CO_2_ generated from reaction R2. The higher reactivity of anthracite coke toward CO_2_ is expected because of its much less ordered carbon structure compared to that of mesophase-derived coke produced at 950 °C. This difference explains the more pronounced effect of porosity reorganization in the case of anthracite. 

##### The Impact of the KOH/Precursor Ratio

**Figure 8:**
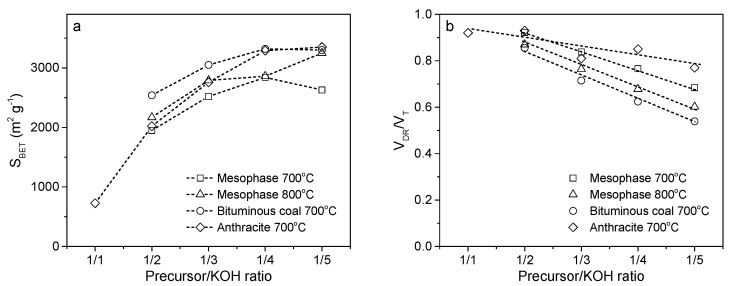
Effect of precursor/KOH ratio on the BET surface area (**a**) and contribution of micropores (**b**) of ACs produced from selected raw materials by KOH activation at 700 and 800 °C for 1 h.

Generally, an excess amount of KOH relative to the reaction stoichiometry should be used in this process. A part of the potassium hydroxide is transferred to less-reactive carbonate, leading to a decrease in the activation rate because of the lower KOH concentration. This finding is supported by the data shown in Figure 8. When pitch mesophase, char from bituminous coal and anthracite are activated at 700 and 800 °C for 1 h at various KOH/precursor ratios, a gradual but non-linear increase in the BET surface areas of the resulting carbons is observed as the KOH/precursor ratio increases from 1:1 to 4:1. However, higher amounts of KOH seem to be useless in terms of porosity development. The activation process is so drastic that previously created porosity is destroyed by reaction with an excess of KOH. This effect seems to be more pronounced at lower process temperatures. In contrast, because the KOH/precursor ratio is related to the process rate, when KOH is added in excess, the activation can occur so fast that noticeably wider pores are created. A nearly linear decrease in the contribution of the micropore volume to the total pore volume is observed as the KOH/precursor ratio increases. Moreover, a higher KOH/precursor ratio promotes the development of pores with higher mean micropore sizes and wider pore size distributions calculated based on the Dubinin-Stoeckli equation [52].

##### The Impact of the Precursor Nature

The nature of the precursor strongly influences porosity development in the KOH activation process. Generally, raw materials with more ordered carbon structures are less prone to the development of porosity during KOH/NaOH activation. For example, the low-rank coals, such as lignite and subbituminous coal, are very reactive towards KOH and NaOH, and thus, ACs with extremely high BET surface areas above 3000 m^2^ g^−1^ but low contributions of micropores are obtained under relatively mild activation conditions [59,60,61,62]. In a similar process, high-rank anthracites can be activated to yield ACs with surface areas of 1900–2800 m^2^ g^−1^ [52,63]. The ACs produced from commercial carbon fibers are highly microporous solids but have SSA values below 2000 m^2^ g^−1^ [54,64]. Well-ordered multi-wall carbon nanotubes (MWCNTs) can be transformed to mesoporous powders with surface areas of 1200–1700 m^2^ g^−1^ via KOH activation [65,66]. Finally, graphite is resistant to this activation agent [55].

The above results prove that the behavior of the precursor during activation is related to its susceptibility to graphitization. The effect is more pronounced when pre-carbonization of the material in inert gas is applied before the heat treatment with KOH, as observed, for example, for cokes/chars prepared from bituminous coal (Table 5) and coal-tar pitch (Table 6)**.**

A higher HTT of coal chars significantly decreases their reactivity with KOH, thereby decreasing their porosity development. Nevertheless, preparing char at 1000 °C facilitates obtaining ACs with BET surface areas of approximately 1000 m^2^ g^−1^. A comparable contribution of the micropore volume (87–93%) was detected in all ACs prepared, but lower porosity development was associated with a significantly lower mean micropore size, L_0_. In the case of pitch coke, a sharp decrease in porosity and increase in L_0_ were observed when the precursor was pretreated above 700 °C. Noticeable precursor burn-off is still detected at pretreatment temperatures of 900 and 1000 °C, but it is not related to porosity development.

Decreasing reactivity was associated with the structure ordering of other graphitizable materials such as MWCNTs [66]. When the precursor was pre-heated at 450, 500 and 600 °C, gradually lower BET surface areas (1670, 1220 and 868 m^2^ g^−1^, respectively) and wider pore size distributions were observed after KOH activation at 800 °C. 

**Figure 9:**
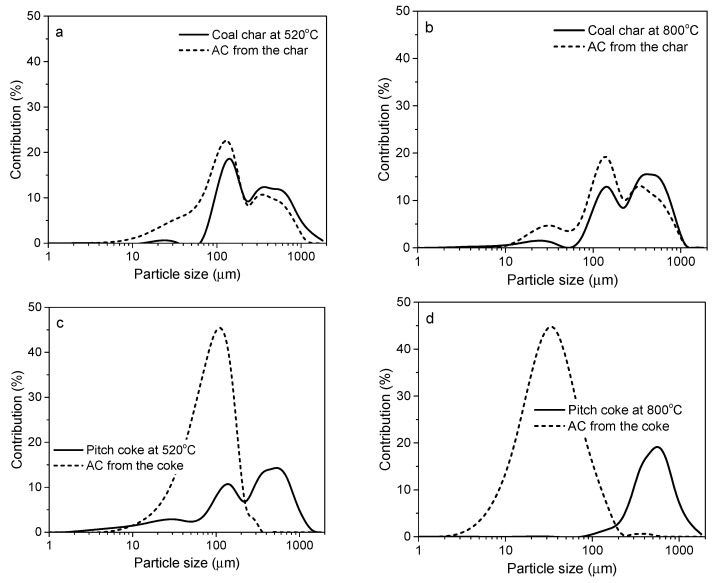
Particle size distribution of the char/cokes from bituminous coal (**a**,**b**) and coal-tar pitch (**c**,**d**) and resultant ACs produced by KOH activation. Precursor/KOH ratio 1:3, reaction temperature 800 °C, soaking 1 h.

These different behaviors of graphitizable and non-graphitizable materials are attributable to the activation mechanism. For well-ordered cokes from pitch or MWCNTs, the potassium intercalation between graphene layers occurs more easily and to a higher extent. The de-intercalation process is then so dynamic that the graphene layer expansion destroys the material integrity. Determining the particle sizes before and after activation confirms this finding (Figure 9). In the HTT range of 520–800 °C, similar particle size distributions were observed for chars from bituminous coal and the resultant ACs. In contrast, the starting grains of pitch-derived coke disintegrated to fine powder. The effect is more pronounced when higher HTT is applied to the precursor.

**Figure 10:**
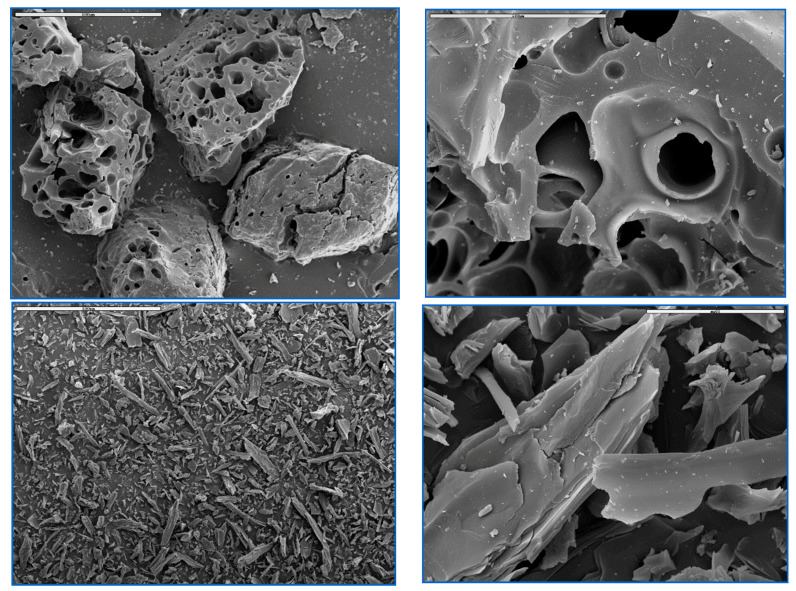
SEM images of ACs produced by KOH activation from bituminous coal (top) and pitch (bottom), pre-carbonized at 800 °C. Precursor/KOH ratio 1:3, reaction temperature 800 °C, soaking time 1 h.

In addition to the average particle size, significant differences in the grain morphologies are also observed (Figure 10). Scanning electron microscopy (SEM) reveals that coal-derived ACs form isometric particles with large macropores, whereas pitch-derived ACs form needle-like fine particles because of the anisotropic nature of the precursor.

##### The Impact of the Reaction Time

**Figure 11:**
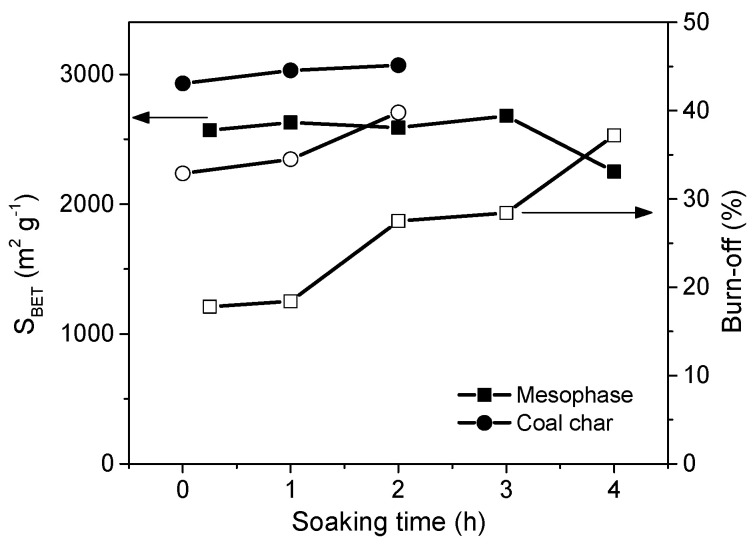
Effect of soaking time on the BET surface area (closed symbols) and burn-off (opened symbols) of coal-tar pitch mesophase and bituminous coal-derived char activated by KOH at a precursor/KOH ratio of 1:3 at 800 °C.

The reactions that occur during alkali activation are very fast. The porosity development of a precursor begins during the heating of the reactor. Further soaking at the desired temperature results in no significant effect, opposite to that observed in physical activation, in which the porosity development of the carbon is almost proportional to the reaction time. Carbons with very high SSAs can be produced by KOH activation after only 1 h of soaking. Several examples are presented in Figure 11. The KOH activation of pitch mesophase facilitates obtaining ACs with BET surface areas of 2600 m^2^ g^−1^ after 15 min of soaking. A longer soaking time results in higher burn-off, and no porosity development occurs. The bituminous coal-based char is more reactive, and as a result, a BET surface area of 2930 m^2^ g^−1^ was obtained immediately after reaching the final HTT. A similar behavior was reported by Linares-Solano et al. [67] for anthracites. After 0.5 h of soaking, the BET surface area attained a value of approximately 1800 m^2^ g^−1^, which increased by only 18% when the process was prolonged to 2 h.

##### The Impact of the Inert Gas Flow Rate 

The influence of the inert gas flow on the KOH activation process has been ignored for many years. The first report was published by Lozano-Castello et al. [67]. They revealed that the flow rate of nitrogen passing through the reactor strongly affects the porous structure of the resultant AC. For a KOH/anthracite ratio of 2:1 and HTT of 700 °C, increasing the gas flow rate from 80 to 800 mL min^−1^ enhances the BET surface area of the resulting AC from 950 to 2020 m^2^ g^−1^. Moreover, replacing nitrogen with a higher-molecular weight gas (here, argon) increased the porosity by 4%, whereas treatment in light helium decreases it by 13%. This finding can be related to the efficiency of the removal of reaction products, such as H_2_ and alkaline metals, and a shift of the activation equilibrium towards the reaction products. Generally, a higher flow rate and higher density of the purge gas enhance the rate of mass transfer. 

##### Other Factors

A simple physical mixture of anhydrous KOH and precursor powders can generally be carbonized satisfactorily because the KOH melts during the heating of the reactor, and a suspension of char particles in liquid KOH is present at the final process temperature. However, before carbonization, lignocellulosic raw materials and other low-carbonized precursors are often impregnated with a water solution of KOH [52,68], which helps to disperse the chemical and facilitates producing a more homogenous AC with higher porosity development.

#### 3.2.2. Phosphoric Acid Activation

**Figure 12:**
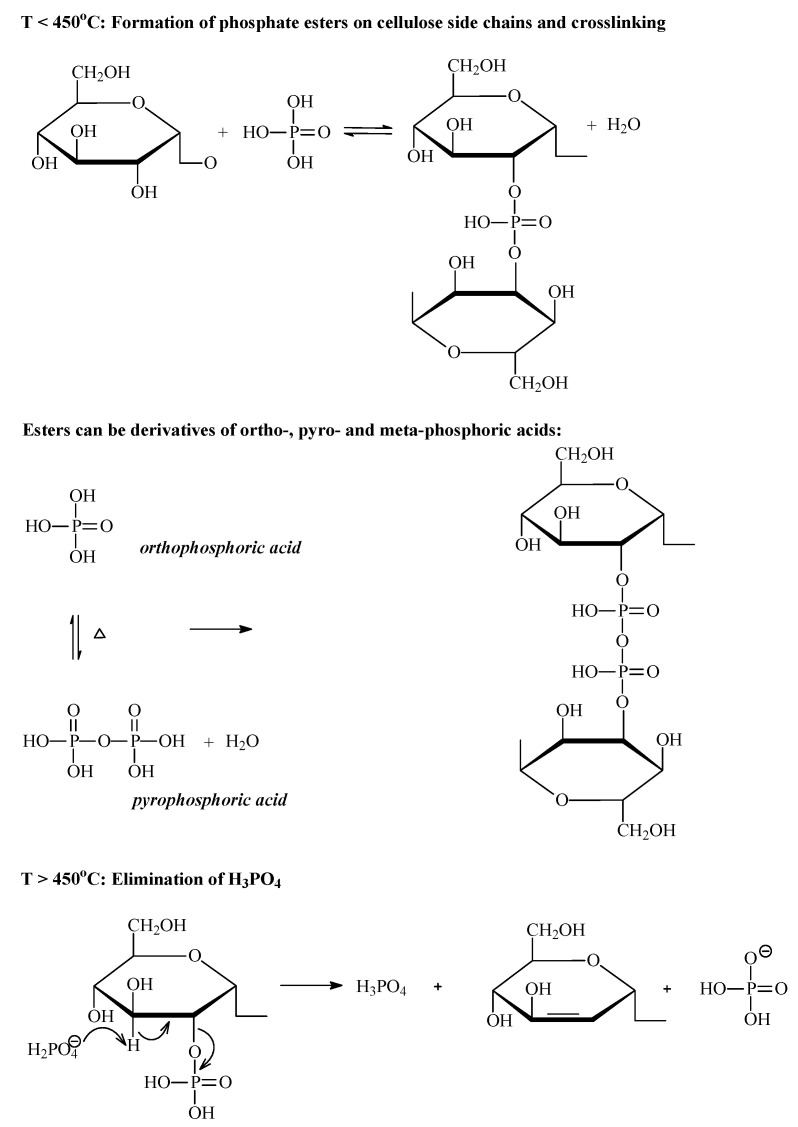
Mechanism of phosphate ester formation by phosphorylation of cellulose. Reproduced from [72] with permission from Elsevier © 1998.

Wood, fruit stones, nut shells and many other lignocellulosic materials are mostly used as raw materials for the preparation of AC using H_3_PO_4_ because of its high reactivity towards biopolymers. The activation of subbituminous and bituminous coals yields materials with relatively low porosity development [69], and polymers [70,71] are more suitable as precursors than coal. A fundamental study on the chemistry of the H_3_PO_4_ activation of wood was performed by Jagtoyen and Derbyshire [72]. H_3_PO_4_ promotes the degradation of biopolymers during heat treatment, and the bond cleavage and depolymerization reactions proceed at temperatures lower than 150 °C. This acid hydrolyzes glycoside linkages in the cellulose and hemicellulose and cleaves aryl-ether bonds in the lignin. Subsequently, dehydration, degradation and condensation occur, leading to an increase in aromaticity, followed by the evolution of CO_2_, CO and CH_4_. In addition, the reaction of H_3_PO_4_ with cellulose is accompanied by inter-crystalline and intra-crystalline swelling. Above 150 °C, cross-linking reactions begin to be more pronounced, as reflected by structural dilatation resulting from the insertion of phosphate groups between the cellulose chains, generating porosity. The mechanisms of the activation of cellulose by H_3_PO_4_, including the formation of ester phosphates, are shown in Figure 12.

This figure illustrates how phosphoric acid can be inserted between the cellulose chains, thereby disrupting the existing hydrogen bonds, replacing them with other chemical linkages, and simultaneously separating the chains and dilating the structure. Phosphate groups act as bridges preventing the contraction of pores. As the temperature increases, small polyaromatic units connected via phosphate and polyphosphate linkages grow via cyclization and condensation reactions, further increasing the aromaticity of the carbon structure [73]. Among the phosphorous species present in the char, pyrophosphate predominates, followed by metaphosphate and phosphate, as revealed by X-ray photoelectron spectroscopy (XPS) [74]. Phosphoric acid acts as an efficient oxidant to form carbon-oxygen bonds, supported by an enhanced oxygen content and the presence of quinone C=O groups, ether C-O-C groups, P-O-C groups, hydroxylic C-OH and carboxylic groups COOH [74,75]. Above 450 °C, the breakdown of phosphate bridges occurs, as reflected by the contraction of the structure, which leads to materials with reduced porosity. 

Further structural rearrangements, including aromatic condensation, increase the size of the aromatic units and the carbon content in the char [74]. At approximately 600 °C, the phosphates disappear, and the phosphorus pentoxide P_4_O_10_ appears in the structure in addition to the remaining pyrophosphates and metaphosphates in the char [75]. To obtain a material with a developed porous structure, the resultant char is washed with water to remove the acid, leaving pores in the expanded carbon matrix. H_3_PO_4_ ACs have hydrophilic and acidic surfaces and exhibit high cation exchange capacities related to the presence of oxygen- and phosphorous-containing surface groups [76,77].

**Figure 13:**
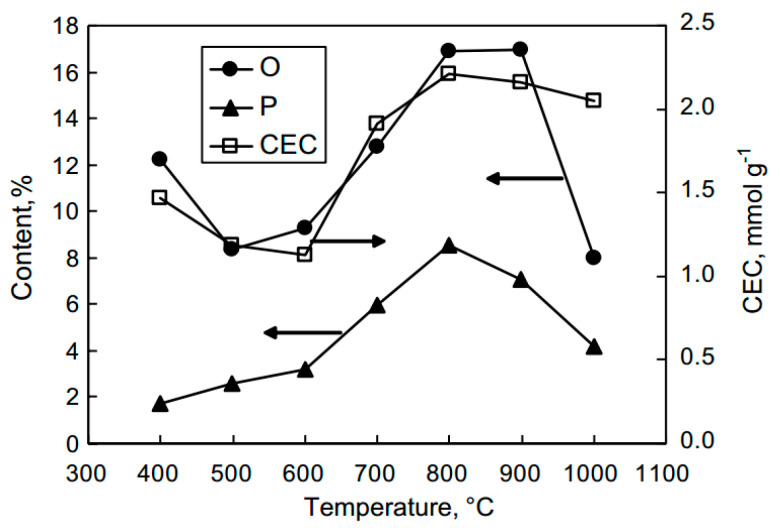
Phosphorous and oxygen contents together with cation exchange capacity (CEC) of H_3_PO_4_ ACs as a function of heat treatment temperature. Reproduced from [78] with permission from Elsevier © 2005.

The changes in the chemical structure during H_3_PO_4_ activation are reflected in the elemental compositions of the resulting carbons. Figure 13 shows the variations of the phosphorous and oxygen contents obtained from fruit stones as the activation temperature is increased [78]. As the temperature increases up to 500 °C, the oxygen content decreases because of the dehydrating effect of phosphoric acid, which is accompanied by a decrease in the phosphorous content. Above this temperature, the carbon structure becomes enriched in both oxygen and phosphorous, with maximum enrichment occurring at 800–900 °C. The volatilization of phosphorous-containing compounds and/or reduction of P_4_O_10_ by carbon accounts for the subsequent decrease in both elements at higher temperatures [79,80]. However, at 1000 °C, the resulting carbon material still contains a large amount of heteroatoms, approximately 8 wt% oxygen and 4 wt% of phosphorous [74]. The phosphorous content is related not only to the activation temperature but also to the amount of activating agent used. Generally, as the amount of H_3_PO_4_ incorporated into a raw material increases, the phosphorous content in the carbon also increases [81].

The advantages of H_3_PO_4_ activation for producing porous materials include high product yields (25–45%) compared with physical activation (15–20%). This advantage is a result of the chemical and structural alternation of the biopolymers by the activating agent via extensive cross-linking, which implies a decrease in the evolution of volatiles and, thus, an increase in the carbon yield [73]. 

Based on the chemistry of activation with H_3_PO_4_ described above, it can be deduced that temperatures between 400 and 500 °C should be optimal for the formation of porous structures. This is related to the expanded carbon matrix at these temperatures, which is highly preserved because of the removal of phosphorous compounds after activation. At higher temperatures, shrinkage of the structure because of the decomposition of phosphate linkages leads to reduced porosity. 

**Figure 14:**
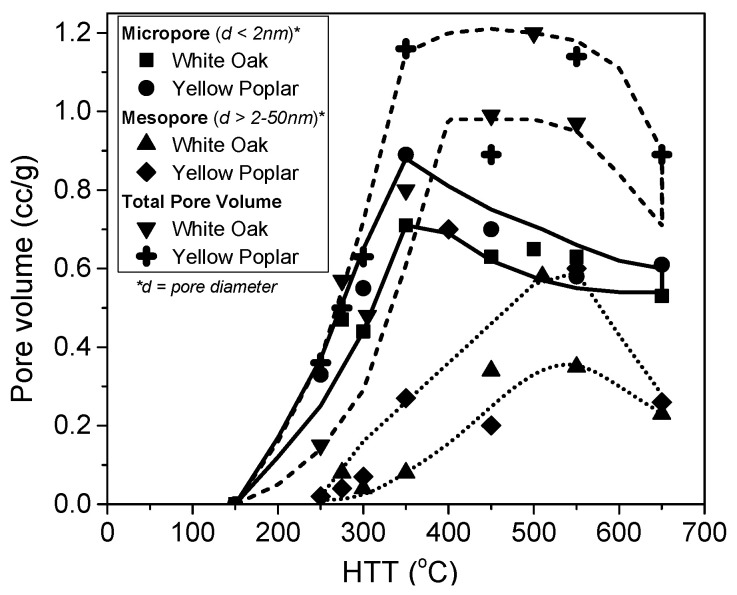
Pore volume of AC from white oak and yellow poplar (H_3_PO_4_/wood= 1.45 g/g, acid strength 28%). Reproduced from [72] with permission from Elsevier © 1998.

Figure 14 shows the changes in the porosity evolution with increasing temperature during the H_3_PO_4_ activation of white oak and yellow poplar [72]. The micropore volume peaks at approximately 350 °C, whereas the mesopore development is shifted to higher temperatures, reaching a maximum at 500–550 °C. Thus, micropore widening contributes to increasing the mesopore volume. As follows from Figure 14, to maximize the micropore volume, a temperature near 400 °C is most appropriate. Heat treatment at higher temperatures promotes the evolution of mesopores. It should be added that depending on the other process parameters, the optimal temperatures for micropore and mesopore evolution can be shifted to some extent.

**Figure 15:**
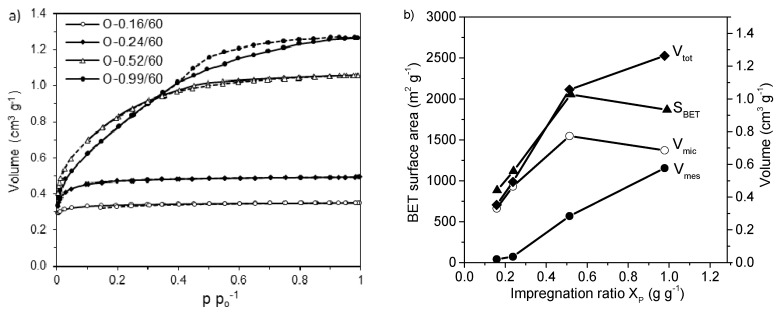
Adsorption (-) and (---) desorption N_2_ isotherms at 77 K (**a**) and variations in the BET surface area (S_BET_), total pore volume (V_T_), micropore volume (V_mic_), mesopore volume (V_mes_) (**b**) for ACs from oak prepared by phosphoric acid activation at different impregnation ratio (g P/g precursor). Reproduced from [4] with permission from Elsevier © 2007.

Other process variables that are highly relevant for porosity development include the amount of activating agent used and, to a smaller extent, the soaking time. Many studies have addressed the influence of the H_3_PO_4_:precursor ratio at a constant soaking time. Generally, regardless of the lignocellulosic precursor, a low impregnation ratio, as defined by the amount of phosphorous or H_3_PO_4_ incorporated per gram of precursor, promotes the evolution of micropores. In contrast, higher impregnation ratios favor the development of wide micropores and mesopores. However, an excess of the activating agent results in decreased microporosity because of the widening or even collapse of the existing pores. These changes in the porosity development are being investigated by many researchers for various lignocellulosic precursors, such as corncob [81], peach stone [82,83], cotton stalks [84], birch [4], hemp stem [9], pecan shells [85], kenaf [86] and eucalyptus residue [87]. For example, the influence of the amount of activating agent used can be clearly observed in the N_2_ adsorption-desorption isotherms measured at 77K for ACs prepared from oak (Figure 15) [4].

As the impregnation ratio increases from 0.16 to 0.99 (g P/g precursor), the shape of the adsorption isotherm gradually changes following the IUPAC classification system from type I, which represents microporous carbon, to type IV, which exhibits adsorption-desorption hysteresis, confirming the high contribution of mesopores in the porous structure. Moreover, the less-sharp knees of these isotherms at low relative pressures indicate that the heterogeneity of the micropore width increases with the impregnation ratio. The differences in the N_2_ isotherms are reflected in the textural parameters calculated from them. As the impregnation ratio increases, the micropore volume and BET surface area increase, peak at an impregnation ratio of 0.52 and then decrease. In contrast, the mesopore volume increases continuously (Figure 15). The same mesopore evolution trend was reported for peach stones during activation with H_3_PO_4_ at 450 °C; however, the micropore volume reached a maximum at a lower impregnation ratio of 0.15 [82].

Comparing the results of studies on the impact of the amount of H_3_PO_4_ on porosity development reported by different authors is not easy because the amounts are expressed in different forms: acid concentration/g precursor, g acid/g precursor or g P/g precursor. Moreover, the optimal amount of H_3_PO_4_ introduced into a raw material to obtain the highest surface area is related to many process variables, including the impregnation temperature, pretreatment conditions, precursor particle size, activation temperature, time and atmosphere. Therefore, the H_3_PO_4_ activation parameters must be optimized for a given precursor to obtain the desired porous texture characteristic of the resultant AC.

As mentioned above, the activation time affects the porosity development to a smaller extent than the impregnation ratio and activation temperature. A reasonable effect of soaking time on the structural parameters of ACs can be observed for short activation times, as reported for carbons produced from hemp stem [9] At an impregnation ratio of 0.52 g P/g precursor, with increasing soaking time from 10 to 30 min, the BET surface area increases, reaches a maximum value of 2215 m^2^ g^−1^, and then decreases because of the destructive influence of longer soaking times on the development of both micropores and mesopores. However, in most studies, the soaking time at the final activation temperature is 1 or 2 h. 

**Figure 16:**
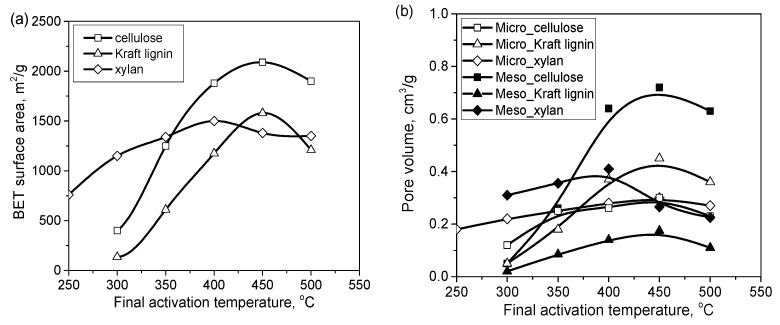
Effect of final activation temperature on (**a**) BET surface areas and (**b**) pore volumes of carbons produced from xylan, cellulose, and Kraft lignin at an impregnation ratio of 1.5. Reproduced from [88] with permission from Elsevier © 2006.

The porosity development during H_3_PO_4_ activation depends on the composition of the lignocellulosic materials used as the precursor. Their compositions, which mainly include cellulose, lignin and hemicellulose, vary between plant species [1]. For example, hemp stem contains over 70 wt% cellulose, whereas palm kernel shell contains approximately 21 wt%. The biopolymers are differentiated in terms of their porosity development during H_3_PO_4_ activation. As shown in Figure 16, for both cellulose and lignin, the volumes of micropores and mesopores are maximized at 450 °C and then decrease as the activation temperature is increased further [88]. The BET surface areas and micropore volumes of carbons from lignin are higher than those for carbons from cellulose and xylan, whereas the highest mesopore volumes are obtained for the carbons prepared from cellulose at temperatures above 350 °C. These findings confirm the mechanism of H_3_PO_4_ activation through the formation of phosphate linkages between the cellulose chains [72]. The differences in the biopolymer behaviors are related to their constitutions. The space between the crystalline cellulose microfibrils in an array is on the order of a few nanometers, similar to small mesopores. The ordered structure of crystalline cellulose has a high propensity that binds phosphoric groups through reactions with hydroxyl groups, resulting in structure expansion [72]. Because of its amorphous nature, lignin shows a significantly lower potential for expansion, resulting in narrower porosity [72,89]. Indeed, the cellulose-rich precursors yield carbons with higher contributions of mesopores than lignin-enriched precursors when the same impregnation ratio and activation conditions are used [4,72,73]. 

In most reports, the activation process using H_3_PO_4_ was performed in an inert atmosphere. Indeed, the gaseous atmosphere can also affect the porous structure of the resultant AC. CO_2_ [90,91,92], air [89] and steam [4,75,89] were applied to modify the conditions for porosity development. Interestingly, the use of steam yields a product with enhanced mesoporosity and lower content of heteroatoms than were obtained using nitrogen alone. For example, the oxygen content decreased from 20.5 to 8.2 wt% for carbons prepared from oak by H_3_PO_4_ activation at 480 °C, when steam was introduced into the nitrogen stream at 300 °C [4]. Steam is believed to inhibit the formation of condensate phosphates because of the protogenic character of H_3_PO_4_, which facilitates the elimination of phosphate groups that were previously incorporated in the carbon structure [75,89]. It is important to emphasize that the use of steam during H_3_PO_4_ activation facilitates obtaining highly mesoporous carbons with mesopore contributions over 60%, low oxygen contents and very low phosphorous contents (<0.4 wt%), which cannot be achieved when the process is performed under a flow of inert gas. 

In summary, the activation of lignocellulosic precursors with H_3_PO_4_ has great potential as a method for easily tailoring the textural parameters of the resultant ACs over a wide range from highly microporous to mesoporous materials with different pore size distributions. Depending on the biomass precursor and process conditions, H_3_PO_4_ activation provides microporous materials with BET surface areas of up to 2500 m^2^ g^−1^ and micropore pore volumes of up to 0.95 cm^3^ g^−1^. However, compared with KOH activated carbons, H_3_PO_4_ activated carbons are characterized by wider and more heterogeneous microporosity. As mentioned above, ACs with high mesopore volumes (i.e., over 1.5 cm^3^ g^−1^) can also be produced by adjusting suitable parameters of the H_3_PO_4_ activation process. Moreover, this strategy allows the utilization of agricultural by-products and wastes, thereby decreasing the cost of AC production. Despite the very low yields obtained in some cases, the ACs produced may be attractive as electrode materials for supercapacitors. 

Table 7 shows selected biomass waste-based ACs obtained by activation with H_3_PO_4_, their activation parameters and the characteristics of their porous structures. 

#### 3.2.3. Zinc Chloride Activation

ZnCl_2_ is another chemical agent in addition to H_3_PO_4_ and was widely used up to the 1970s for the production of porous carbons from lignocellulosic materials. Currently, the industrial-scale production of ACs by ZnCl_2_ activation is limited, but this method is still used by researchers in their laboratories. This process was replaced by H_3_PO_4_ activation because of the limited efficiency of ZnCl_2_ recovery, problems with corrosion, environmental disadvantages and the presence of residual zinc in the AC. Lignocellulosic precursors are preferred for the production of porous carbons by ZnCl_2_ activation. Similar to H_3_PO_4_, ZnCl_2_ is a strong dehydrating agent that promotes the biopolymer degradation. Precursor particles impregnated with an aqueous solution of ZnCl_2_ are subjected to heat treatment in an inert atmosphere at temperatures of 400–900 °C. The mechanism of ZnCl_2_ activation was reported by Caturla et al. [97]. During impregnation, ZnCl_2_ penetrates the interior of the precursor particles, leading to some hydrolysis of the biopolymer components and swelling of the particles. The dehydration of the precursor followed by aromatization during heat treatment results in structural contraction but only to a small extent because the reactant remains inside the particles, serving as a template for porosity creation. As for H_3_PO_4_, washing the heat-treated particles with water results in a porous material. The mechanism of the evolution of porosity during ZnCl_2_ activation was confirmed by comparing the volumes of micropores and mesopores in the resultant carbon with the volume of ZnCl_2_ used for impregnation. 

Generally, ZnCl_2_ is most effective in the development of micropores at temperatures of 500–600 °C. Higher temperatures result in contraction of the particles, accompanied by decreased microporosity. No further reaction of ZnCl_2_ with the carbon seems to proceed above 500 °C [98]. However, for some precursors, a reasonable increase in the micropore volume was reported at temperatures higher than 600 °C [99,100]. These results emphasize the variety of factors, including the impregnation conditions and experimental set-up, that determine the porous structure of the final product. However, in most reports on the activation of different precursors with ZnCl_2_, the activation temperature was chosen arbitrarily. The changes in the carbon texture during the heat treatment of ground coffee impregnated with ZnCl_2_ were monitored in situ during thermal treatment up to 750 °C by environmental SEM (ESEM) [99]. The most remarkable changes in the texture were observed between 150 and 340 °C because of the removal of volatile matter produced by the decomposition of the precursor, followed by shrinkage and cross-linking reactions in the carbon matrix. 

**Figure 17:**
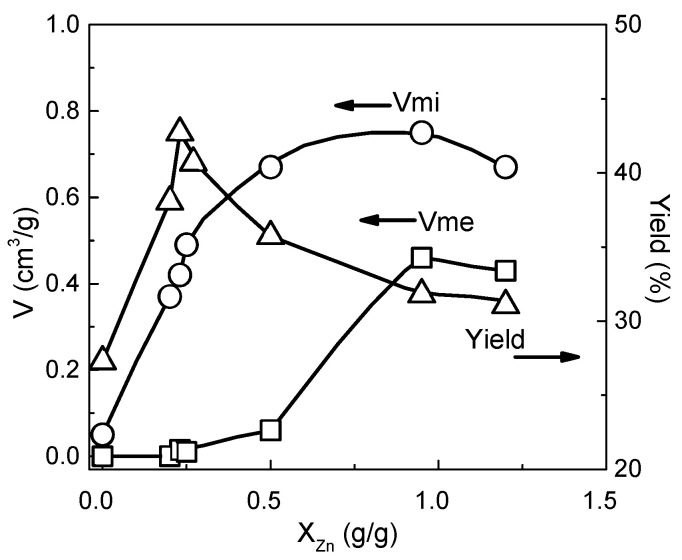
Influence of the impregnation ratio on the porosity development and carbon yield during activation of peach stones using ZnCl_2_. Circle (V_mi_) – micropores volume, square (V_me_) – mesopores volume, triangle – process yield. Reproduced from [83] with permission from Elsevier © 2004.

Apart from the HTT, the most important variable in the ZnCl_2_ activation process that determines the porous structure of the resulting AC is the amount of the activating agent introduced into the precursor during impregnation. The influence of the impregnation ratio on porosity development during ZnCl_2_ activation has been studied for a wide range of lignocellulosic materials [100,101,102,103,104,105,106]. For example, the relationships between the impregnation ratio (g Zn/g precursor), the volumes of micropores and mesopores and the yield of carbon obtained at 500 °C from peach stones are shown in Figure 17.

At a low impregnation ratio (less than X_Zn_ = 0.4 g g^−1^), the development of micropores is very fast. The micropores are narrow and have uniform sizes that are related to the small size of the ZnCl_2_ molecule or its hydrates. As the impregnation ratio increases further, micropores continue to be developed but at a slower rate and with a more heterogeneous size distribution. The volume of these pores is maximized at X_Zn_ = 1 g g^−1^ and corresponds to a BET surface area of over 2000 m^2^ g^−1^. The increase in the microporosity is accompanied by the development of mesopores, which is particularly intensive at X_Zn_ > 0.4 g g^−1^ and is reflected by a decrease in the carbon yield. At a high impregnation ratio (X_Zn_ > 1 g g^−1^), a significant reduction in both micropores and mesopores is related to the widening of the pores and their subsequent degradation [83,97]. Thus, as the degree of impregnation increases, the average pore size of the resulting AC also increases. Generally, the highest micropore volume and surface area are obtained at temperatures of 500–600 °C at a ZnCl_2_/precursor ratio of 1.5–2.0. For example, the BET surface area of macadamia nutshell-derived ACs reached a maximum (approximately 1600 m^2^ g^−1^) when the process was performed at 500 °C with a ZnCl_2_/precursor ratio of 2, which corresponds to X_Zn_ = 1 g g^−1^ [107]. In contrast, a maximum BET surface area of over 1200 m^2^ g^−1^ was obtained for almond shells impregnated at the same impregnation ratio but subjected to higher temperature (i.e., 600 °C) [108]. At high impregnation ratios of 4–6, mesoporous ACs with developed surface area were obtained at temperatures of 600–700 °C [109,110]. Compared to the amount of the activating agent used, the activation time has a smaller effect on the development of microporosity. One to two hours of carbonization seems to be optimal. 

The activation process parameters, such as the amount of the activating agent and the activation temperature, have an influence on the elemental composition of the resultant carbon. With increasing activation temperature and impregnation ratio, the carbon content increases followed by a decrease in the oxygen content. For example, the oxygen content in the cherry stone-derived activated carbon decreased from approximately 24 to 14 wt%, as the temperatures increased from 500 to 900 °C [99]. A decrease in the oxygen content from 29 to 15 wt% was observed with increasing the impregnation ratio from 1:1 to 4:1 for the same precursor activated at 700 °C. 

In the recent decade, the preparation of low-cost porous materials from agricultural and industrial waste biomass using ZnCl_2_ has been a growing interest. Various precursors, such as cashew nut shell [111], cherry stones [100,104], Fox nut shell [101], grape stalk [102], palm shell [112], rice husk [113], sugar cane bagasse [114], ground coffee [99], willow leaves [115], corncob [103], chestnut shell [116], peanut shell [113], oak cups pulp [117] and hemicellulose [118] were used for this purpose. The obtained low-cost porous materials are mainly considered as adsorbents for removal of contaminants from water, but also there are attempts to use them as electrode material for supercapacitors [113,114,115,116,117,118,119,120]. Depending on the precursor and the activation process parameters, a wide range of activated carbons have been prepared by ZnCl_2_ activation. They differed by textural parameters, such as the specific surface area, total volume and pore volume distribution. The microporosity of ZnCl_2_ activated carbons is more uniform in the terms of the pore size than that developed by H_3_PO_4_. However, it is wider than that of KOH activated carbons. 

Table 8 shows the textural characteristics of selected activated carbons prepared from different precursors by ZnCl_2_ activation and the process conditions in which they were obtained. Considering the ZnCl_2_ activated carbon as electrode material for supercapacitors, the residual content of Zn should be taken into account as it can be detrimental for the capacitor performance working in organic electrolyte, which will be discussed in the Section 4.4.

### 3.3. Self-Activation

The term “self-activation” is sometimes used to explain the above-average development of porosity occurring during high-temperature pyrolysis of organic materials. Some gases released during the thermal decomposition of raw material (mainly CO_2_ and H_2_O) can react with the carbon residue on the basis of physical activation rules [124,125]. However, more often this term is used for clarification the processes occurred during pyrolysis of a precursor containing a high amount of alkali elements chemically bonded to the carbon structure. The porosity development is controlled by the same reaction as in conventional chemical activation. In self-activation, raw materials containing potassium or sodium are preferred. Because of the perfect elemental dispersion at the molecular level, the pyrolysis of a precursor with relatively low mineral matter contents can produce a highly porous AC. A wide range of synthetic precursors, such as potassium salts of high-molecular weight organic acids, can undergo self-activation. However, for large-scale production, biomass has been proposed as a cheap raw material for this process.

Raymundo-Piñero et al. [126] suggested obtaining porous carbons by the simple pyrolysis of seaweed, a biomass containing sodium alginate biopolymer. This process was performed under a nitrogen atmosphere between 600 and 900 °C. The porosity development of the product was strongly dependent on the origin of the precursor, exhibited a wide range between 14 and 750 m^2^ g^−1^ at 600 °C, and showed a micro/mesoporous texture. Higher treatment temperatures facilitated achieving materials with better-developed porosity (BET surface areas up to 1300 m^2^ g^−1^) but higher contributions of mesopores (up to 50%). The pyrolysis of isolated, pure sodium alginate can also be used as an alternative to non-defined seaweed, resulting in a similar effect on the porosity development of the product [127].

Recently, tobacco stems were suggested as a raw material for self-activation [128]. Tobacco stems are a waste product from the tobacco industry that contain over 10 wt% of alkali elements, mostly potassium. As expected, the properties of the carbonization product depend on the origin of the precursor. The pyrolysis of tobacco stems at 600 °C can produce carbons with relatively low BET surface areas (320–810 m^2^ g^−1^). At 900 °C, the porosity can increase up to 1440 m^2^ g^−1^ (Burley tobacco). In contrast to seaweed, carbons with very narrow micropores (L_0_ = 0.6–1.1 nm) can be produced from tobacco stems. 

In summary, the results of self-activation depend strongly on the precursor chosen, and self-activation is usually less effective than conventional chemical activation. Moreover, in both cases, the process must be conducted in a reactor that is resistant to alkaline vapor, and the product must be washed/demineralized with acid before further application. 

### 3.4. Catalytic Activation

**Figure 18:**
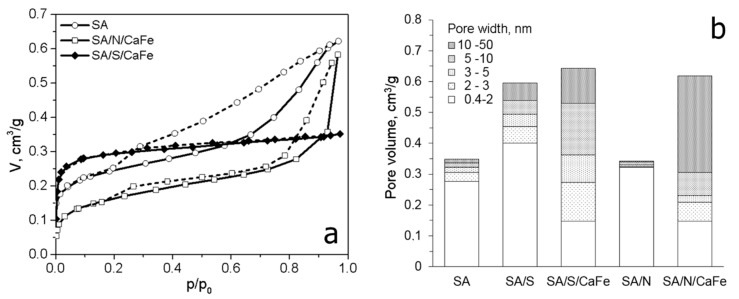
Adsorption isotherms of benzene at 25 °C (**a**) and pore volume distribution (**b**) of ACs prepared from bituminous coal. SA-AC from bituminous coal; SA/N/CaFe and SA/S/CaFe—AC produced from the oxidized coal (N) and sulphonated (S), respectively, and subsequently loaded with Ca and Fe by ion exchange. Solid lines—adsorption branch, dashed lines—desorption branch. Reproduced from [130] with permission from Elsevier © 2004.

Physical activation using gaseous activating agents provides microporous carbons. The introduction of a catalyst into a carbon precursor can lead to the development of mesopores. The reaction of carbon with steam, CO_2_ or molecular oxygen is catalyzed by elements of Groups I and II and transition metals [98]. In these reactions, the catalyst acts as an oxygen carrier. The activating agent molecules preferentially dissociate on the surface of the catalyst to provide mobile atomic oxygen species that can move to the carbon surface to form carbon monoxide and carbon dioxide. Highly mesoporous ACs have been obtained by catalytic gasification from lignite [129], subbituminous and bituminous coals [129,130,131,132], coal-tar pitch [133,134] and petroleum pitch [135]. The efficiency of a catalyst for mesoporosity development depends on the amount of metal introduced into the precursor and the size and distribution of the catalyst particles within the carbon. The metal distribution is closely related to the loading method applied. In most cases, the impregnation of precursor particles with an aqueous solution of appropriate metal salts or with organometallic compounds has been performed. Compared to impregnation, loading by ion exchange leads to materials with more homogeneously dispersed metals, resulting in more developed mesoporosity. In this case, the amount of metal introduced depends on both the content of ion-exchangeable groups in the initial material and the conditions of the ion-exchange process. ACs with mesopore fractions of 77–84% and mesopore volumes of 0.40–0.56 cm^3^ g^−1^ were produced from Ca- and Fe-loaded subbituminous and bituminous coals by ion exchange [129,130]. The cation exchangeability of the coals was achieved by their oxidation or sulfonation. Prior to steam activation, the coals were loaded with iron via the ion exchange of calcium-preloaded coal to avoid the precipitation of iron hydroxides. The adsorption-desorption isotherms of benzene at 25 °C for the ACs prepared from bituminous coal and Ca/Fe-loaded coal are shown in Figure 18a. In this figure, N and S correspond to oxidation with nitric acid and sulfonation with sulfuric acid, respectively. The shapes of the isotherms indicate the creation of different mesoporosity upon activation depending on the method used to enhance the ion-exchange capacity of the initial coal. Sulfonation with subsequent Ca and Fe loading results in the predominant development of mesopores with pore widths of 2–10 nm, whereas the loading of HNO_3_-oxidized coal favors the evolution of larger mesopores in the range of 10–50 nm (Figure 18b). The removal of calcium and iron compounds from the AC after activation increases the mesopore volume by 15%. 

**Figure 19:**
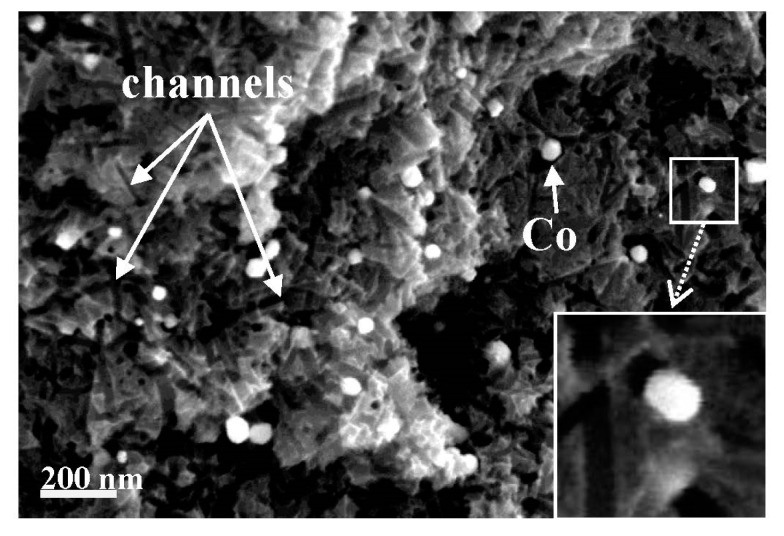
SEM image of Co-ACF and FESEM image of its cross-section surface (before removal of cobalt nanoparticles. Inset: amplification). Reproduced from [137] with permission from Elsevier © 2014.

Porous carbon materials with mesopore fractions above 70% were obtained by the steam activation of petroleum pitch loaded with 1–3 wt% rare-earth metal complexes (i.e., Ln(C_5_H_5_)_3_ and Ln(acac)_3_ [Ln = Sm, Y, Yb, Lu]) [136]. Spherical ACs with developed mesoporosity were obtained from petroleum pitch loaded with Fe, Ni, Co and Y inorganic salts [133] and ferrocene [130]. Catalytic gasification was also successfully applied for the generation of mesopores in pitch-derived carbon fibers [135,136,137,138] and phenolic resin-derived carbon fibers [139], which are microporous in nature. Prior to spinning, the raw materials were loaded with transition metals, such as Co, Al, Ag, Pd, Y, Sm, Yb and Lu. Highly mesoporous AC fibers were prepared from an isotropic anthracene oil-based pitch loaded with cobalt naphthenate and activated with steam [137]. After the removal of Co-containing particles, the BET surface area was nearly 1400 m^2^ g^−1^, and the mesopore volume increased to 0.536 cm^3^ g^−1^. Field emission SEM (FESEM) of the cross-section of Co-activated carbon fiber (Co-ACF) before acid washing shows the channels created during catalytic activation by homogeneously distributed cobalt nanoparticles (Figure 19). 

The sizes of the catalyst particles are comparable to the widths of the mesopores. This result is in agreement with the activation mechanism suggested by Marsh et al. [140], whereby metal particles catalyze gasification in their vicinity via pitting or channeling processes, resulting in the development of mesopores and macropores. Interestingly, alongside the catalytic formation of mesopores, non-catalytic gasification occurs, leading to micropore development. It should be added that the rate of carbon gasification in the presence of a catalyst is enhanced significantly because of the enhanced rate of formation of the chemisorbed oxygen. Therefore, the tailoring of the porous structure through catalytic activation is highly sensitive and requires careful selection of the process conditions to obtain a porous carbon with the desired porous structure.

## 4. Activated Carbons for EDLC

In the conventional supercapacitors, electric energy is accumulated on the electrode surface in the EDL. The specific capacity of the electrode is proportional to the surface accessible to the electrolyte ions. To achieve a high capacity, ACs are commonly used as an electrode materials because of their very high SSAs, relatively good electrical conductivity and low cost.

The SSA is the most important parameter of ACs in supercapacitor applications. However, other factors, such as the pore size distribution, carbon structure ordering, surface chemistry, particle size distribution and pollutants, cannot be ignored because they affect the power density and long-term stability of the cell. 

### 4.1. The Role of the Porous Texture

In the literature, the BET surface area is most often used to characterize the porosity development of ACs. The specific capacity of ACs is almost linearly related to the BET surface area over a wide range of porosities. Depending on the material origin and electrolyte type, the capacitance/S_BET_ ratio is 0.09–0.12 F per square meter. However, above 2000 m^2^ g^−1^, significant perturbations are observed, and the measured specific capacity is often lower than that deduced from the trend. This phenomenon can be explained by the shortcomings of the BET model, which is not reliable for highly porous carbons and does not correspond to the real surface area [141]. The DFT model seems to be more reliable as if allows the calculated specific capacity to be related to the electrochemical capacity over a wider range. However, the relationship remains imperfect [142], and two other factors must be considered. The surface probed by gas molecules (mainly N_2_) is not always the surface occupied by the EDL. For ACs with narrow porosity, some of their surface area may be inaccessible to all anions, and the sieve effect can occur. Even if the pore size is larger than the ion size, the mobility of the charge within the pore structure can be limited, and a drastic decrease in capacitor performance may be observed at higher current load. Additionally, the graphene layers are substantially defect filled in highly porous ACs, and many small chaotic basal planes can be found in such materials. This characteristic results in a strong disturbance of the electron transfer across the material. Some of the carbon structure cannot be polarized during the charging stage, and thus, no EDL is built in this location. The electron transfer limitations have not been considered during the porosity analysis relating to the physical sorption of gas molecules.

Based on the facts mentioned above, the development of the carbon surface area above 2500 m^2^ g^−1^ is unsuitable for application in EDLCs. Moreover, the optimization of the pore size distribution is as important as the surface development. Applying an organic electrolyte with a relatively high ion size is essential. The effective ion sizes of commonly used electrolytes are given in Table 9.

Numerous publications have reported the molecular sieving effect in ACs applied as electrode materials in EDLCs. Eliad et al. [143,144,145,146] analyzed the behaviors of three ACs with very small mean micropore sizes of 0.42, 0.51 and 0.58 nm in several neutral aqueous electrolytes of various cation and anion types: Li_2_SO_4_, LiCl, LiNO_3_, LiClO_4_, MgSO_4_, MgCl_2_, CaCl_2_, BaCl_2_ and NaCl. In theory, the porosity of all carbons should be accessible for all non-solvated ions used in that work. However, the small pore sizes of several of the materials could be a barrier for solvated ions (Table 9). Indeed, ACs of L_0_ = 0.58 nm can readily become positively or negatively charged, even when an electrolyte with large bivalent ions (e.g., Mg^2+^ and SO_4_^2−^) is used. Some perturbations were observed for a carbon of L_0_ = 0.51 nm when it was negatively polarized in MgCl_2_ or positively polarized in LiSO_4_. Almost no capacity was found in MgSO_4_ solution. Clearly, an EDL built of large bivalent ions cannot be stored in such restricted micropores in an AC. A stronger sieving effect was observed when an AC of L_0_ = 0.42 nm was polarized in the presence of Ca^2+^, Mg^2+^ or Ba^2+^ bivalent cations or large ClO_4_^−^ monovalent anions. In contrast, when the carbon was immersed in NaCl electrolyte, EDL formation was not restricted in all potential ranges applied. Moreover, the experiments show that because of the high hydration free energy (Table 9), bivalent ions can only enter the carbon pore system with their hydration shields, whereas monovalent ions form EDLs in dehydrated states.

In practice, ACs with such narrow pores are not used as electrode materials in supercapacitors. However, the sieving affect can also be observed in ACs with typical porosity development if they are charged in organic electrolytes [147] or solvent-free ionic liquids [148]. Raymundo-Pinero et al. [147] analyzed cellulose-derived ACs with DR surface areas in the range of 770–1250 m^2^ g^−1^ and L_0_ values in the range of 0.65–1.18 nm. The materials were charged in an electrolyte consisting of tetraethylammonium tetrafluoroborate (TEABF_4_) in acetonitrile. Only the carbons with L_0_ values higher than 0.80 nm showed proper charge propagation throughout the voltage window. Significant deformations of the cyclic voltammetry (CV) curves occurred when electrode materials with narrower pores were used. A similar behavior was found in the same electrolyte by Mysyk et al. [149]. In a series of ACs with L_0_ values ranging from 0.72 to 1.39 nm, only materials with pores larger than 0.86 nm demonstrated unrestricted EDL charging. 

Comparable results were reported by Ruiz et al. [150] for a series of KOH ACs from PFA. The L_0_ of the ACs ranged from 0.6 to 1.6 nm. These authors analyzed the carbons’ specific capacities in the same electrolyte: 1 mol L^−1^ TEABF_4_ in acetonitrile. Only materials with a mean micropore size equal to or greater than 0.8 nm showed good capacitance behavior throughout the voltage window.

The sieving effect also occurs in ionic liquids in which the ion size ranges from 0.64 to as high as 2 nm. However, in these liquids, the phenomenon is less noticeable than in other types of electrolyte because of the strong anisometric size of the cations. The long carbohydrate chains switch from parallel to perpendicular orientations relative to the carbon surface as they move through the pore system to form EDLs [148]. Moreover, the high viscosity of these electrolytes also affect the rate of charge transfer, and a significant contribution of wider pores is required to ensure adequate performance of ionic liquid-based supercapacitors [151,152]. 

The effective ion size is also dependent on the electrolyte solvent. Decaux et al. [153] analyzed the specific surface capacity of coconut shell-derived CO_2_-activated ACs in several organic electrolytes The BET surface areas of the carbons were 800–1930 m^2^ g^−1^, and the L_0_ values were 0.7–1.5 nm. When TEABF_4_ in acetonitrile was applied as the electrolyte, all electrode materials showed good capacity behavior over a wide range of charge/discharge current densities. The specific surface capacity decreased drastically when the electrolyte was bis(trifluoromethane)sulfonamide lithium salt (LiTFSI) in a mixture of organic carbonates and the electrode materials were ACs with L_0_ values lower than 0.9 nm. These results were somewhat surprising because the unsolvated Li^+^ or TFSI^−^ ions are smaller than TEA^+^ or BF_4_^−^. The authors explained the phenomenon based on the different solvation energies of the ions depending on the electrolyte. Briefly, for TEABF_4_ in acetonitrile, the ions can enter the pores in an unsolvated state, whereas Li^+^ or TFSI^−^ can only be transported with a shield of solvent molecules, mostly ethylene carbonate.

Finally, it must be noted that for thermodynamic reasons, the ion size cannot be *exactly* equal to the pore width. Wider pores support ion mobility and sufficient charge propagation in supercapacitors. However, excessively large pores can be ineffective for the formation of highly dense EDLs. Experimental evidence supporting this statement was provided by Raymundo-Pinero et al. [154]. They analyzed the effective mean micropore size (L_0_) in a series of ACs produced from bituminous coal by KOH activation at 800 °C. The materials were charged in three electrolyte types: 1 mol L^−1^ H_2_SO_4_, 6 mol L^−1^ KOH and 1 mol L^−1^ TEABF_4_ in acetonitrile. The L_0_ ranged between 0.6 and 1.5 nm. The optimal mean micropore sizes were determined to be 0.7 and 0.8 nm in aqueous and organic electrolytes, respectively. The optimum value is approximately 20% higher than the effective ion size with respect to the hydration of bivalent ions (Table 9). A decrease in the efficiency of EDL formation was observed for carbons with higher or lower L_0_ values.

The contribution of mesopores should not be completely ignored because they act as ion transportation routes. This function is more relevant when an organic electrolyte with low ion mobility is applied or a high-power supercapacitor is designed [155,156,157,158]. However, a 10–20% contribution of mesopores to the total porosity of an AC is sufficient to ensure a high charge/discharge rate of the electrode material in commonly used electrolytes.

To summarize, ACs with SSAs of 2000–2500 m^2^ g^−1^ and mean micropore sizes of 0.7–0.8 nm and 0.8–0.9 nm are recommended for most aqueous and organic electrolytes when the gravimetric capacity of the supercapacitor is a key factor. However, from a practical perspective, the volumetric capacity of the final device is also important. In this case, ACs with BET surface areas in a lower range (1500–1800 m^2^ g^−1^) but with higher tap densities have been identified as optimal in several industrial reports [159,160,161]. 

### 4.2. The Role of Structure Ordering

As mentioned above, EDL formation during charging is induced by carbon surface polarization. Thus, easy electron transfer inside the carbon structure is an important factor that influences the performance of the final supercapacitor. Higher carbon structure ordering is known to ensure better electron conductivity in the material. Unfortunately, ACs with high surface areas show relatively low structure ordering. However, the arrangement of the graphene layers in the precursor can be preserved to some extent, even during harsh activation and can, thus, significantly influence the capacity behavior of the resultant material. Qu [162] proved that the specific surface capacitance of an AC is almost proportional to the aspect ratio of the stacking number of graphene sheets (L_c_) to their lateral extent (L_a_), as derived from an X-ray diffraction analysis of ACs.

**Figure 20:**
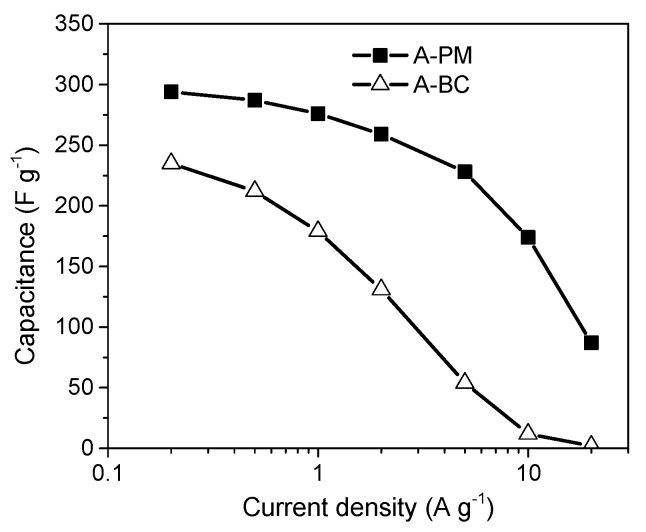
Comparison of the rate capability of ACs produced by KOH activation from pitch mesophase (A-PM) and bituminous coal (A-BC).

This relationship is evident when examining two different raw materials—coal-tar pitch mesophase (graphitizable) and bituminous coal (non-graphitizable)—chosen as AC precursors. In both cases, pre-carbonization at 520–600 °C followed by KOH activation under fixed conditions (800 °C for 1 h, 4:1 KOH/precursor ratio) allowed obtaining ACs with similar BET surface areas of approximately 2600 m^2^ g^−1^, L_0_ values of roughly 1.35 nm and oxygen contents of approximately 6.5 wt%. However, different specific capacities were measured in 6 mol L^−1^ KOH at 0.2 A g^−1^ for mesophase- and coal-derived ACs (294 F g^−1^ [11.0 µF cm^−2^] and 235 F g^−1^ [9.2 µF cm^−2^], respectively). Moreover, a significantly enhanced rate capability was recorded for ACs obtained from better-ordered precursors (Figure 20). This behavior can be explained only by the different conductivities of the electrode materials. 

**Figure 21:**
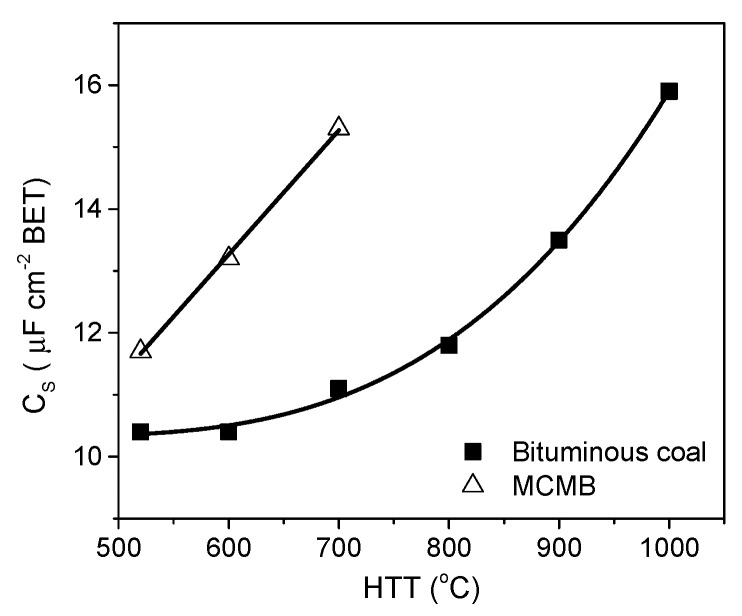
Relation between the specific surface capacity (normalized to BET surface area) of ACs produced by KOH activation at 700 °C and the HTT of different precursors: bituminous coal and mesocarbon microbeads. Reproduced from [163] with permission from Elsevier © 2012. Electrochemical measurement in 6 mol L^−1^ KOH.

Given that the HTT is a strong factor influencing the structure ordering of carbon materials, the higher charge stored on the surface of ACs produced from precursors pre-carbonized at a higher temperature is not surprising. As mentioned previously, a higher carbonization degree of the precursor always leads to lower reactivity toward the activation agent, resulting in lower porosity development in the product. In this case, only comparing the capacity normalized to the surface area of the carbon is reliable. A typical relationship between the HTTs of the selected raw materials and the specific surface capacity (Cs) of the ACs produced by KOH activation is shown in Figure 21. The BET surface areas of ACs derived from mesocarbon microbeads (MCMBs) and bituminous coal were in the ranges of 1830–2640 m^2^ g^−1^ and 800–2740 m^2^ g^−1^, respectively. The activation process was performed under the same conditions to ensure comparable surface chemistries of the ACs. The products of non-graphitizable coal activation show an exponential relationship between Cs and HTT.

In contrast, the AC obtained from graphitizable MCMB pre-carbonized at 520 °C exhibits noticeably better surface charge density (by 13%) than the corresponding coal-derived electrode material. Moreover, the Cs of MCMB-derived ACs depends strongly and almost linearly on the precursor HTT. Therefore, heat treatment of the two raw materials at 700 °C produces ACs with similar BET surface areas (approximately 1850 m^2^ g^−1^), but the MCMB-derived electrode material shows markedly higher Cs (by approximately 40%). Kim et al. [164] compared the capacity properties of a series of KOH AC fibers obtained from graphitizable mesophase and non-graphitizable isotropic pitch. Before the activation process, pre-carbonization of both raw materials was performed at 650 °C. For most ACs, the specific surface capacity measured in an electrolyte of 1 mol L^−1^ TEABF_4_ in propylene carbonate was 50–70% higher if well-ordered mesophase-derived coke was chosen as the precursor. The differences were lower for BET surface areas above 1600 m^2^ g^−1^ because severe activation leads to deeper changes in the carbon structure.

**Figure 22:**
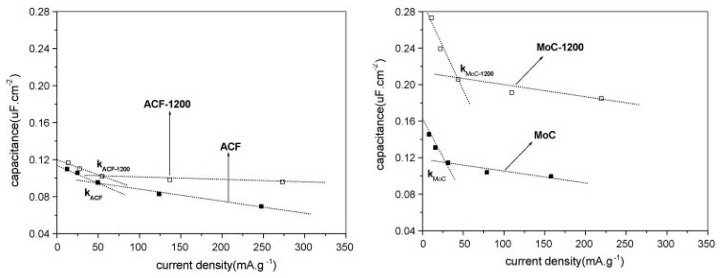
Rate capability of as-received and heat-treated porous carbons of different pore size distribution: ACF—69% of mesopores, MoC—87% of mesopores. 30% KOH as an electrolyte. Reproduced from [167] with permission from Elsevier © 2008.

The structure ordering of ACs can also be enhanced by subjecting the activation product to thermal treatment. However, this procedure always reduces the SSA significantly because of the decomposition of the surface functionalities and widening of the pore system [165,166,167]. Tian et al. [167] reported that this effect is more pronounced when the treatment is applied to ACs with higher contributions of mesopores to the total porosity and in regions of lower current density (Figure 22).

However, ACs with well-ordered carbon structures have one drawback in organic electrolytes: The insertion of solvent molecules (mainly propylene carbonate) between graphene sheets can occur [168,169]. This phenomenon is always related to the swelling of the carbon particles, which leads to the mechanical disintegration of the electrode. 

The above results show clearly that the electric conductivity of activated carbon, resulting from the carbon structure ordering, is as important in influencing the supercapacitor performance as its specific surface area. As the structure ordering of the precursor is partially preserved during activation, the heat-treated soft carbons seem to be the best raw materials for obtaining AC for EDLC. Unfortunately, there is a contradiction here. Well-ordered carbons show much less reactivity towards the activating agent. The activation process must then be carried out for a longer time period or under more aggressive conditions.

### 4.3. The Role of Particle Morphology

The particle size distributions and particle shapes of ACs are technologically important factors. Generally, when used in EDLCs, the active material is ground into a fine powder with an average particle size in the range of 4–8 µm. Moreover, an isometric grain shape is preferred. The combination of these features facilitates producing a flexible electrode with an optimal packing density by pressing the powder with a binder to generate a thin layer [170,171]. This procedure ensures a low amount of void space between the particles and the high volumetric capacity of the final device. This protocol is commonly used in research. However, several authors promote using ACF fabrics as binder-less electrode materials for supercapacitors [172,173,174,175]. Indeed, ACFs have a number of advantages over powdered ACs (PACs). The narrow fiber diameter (10–20 μm) and the opening of micropores directly to the outer surface facilitate access by electrolyte ions. Moreover, the problem of blocking a portion of the parent porosity by a binder does not occur. 

However, a serious drawback of ACFs is their low bulk density (∼0.35 g cm^−3^) relative to the packing density of PACs (0.5–0.7 g cm^−3^) [176]). This low bulk density results in low volumetric capacity, excess electrolyte in void spaces and high electrical resistances between individual fibers and between the fabric and the metallic current collector [177]. To minimize these drawbacks, the fibers can be pulverized to form anisomeric particles. Indeed, an electrochemical investigation clearly revealed the superior behavior of the resultant materials relative to conventional ACs [178,179,180,181].

Wang and Teng [178] produced anisometric particles from commercial PAN-based AC fibers (sample “CF”) and compared their electrochemical properties with those of a laboratory-made isometric AC obtained by the carbonization followed by CO_2_ gasification of phenol-formaldehyde resin (sample “CP2”). Both materials had particle sizes of 10–40 μm and similar BET surface areas of approximately 1300 m^2^ g^−1^. They were heat treated at 900 °C in helium to eliminate the surface groups. CF showed a significantly higher specific capacity (230 F g^−1^) than CP2 (165 F g^−1^) at a low current load in 4 mol L^−1^ H_2_SO_4_. The superiority of the fiber-derived material over the powder was enhanced at a higher discharge rate, despite a lower L_0_ (1.3 vs. 1.6 nm, respectively). The authors attribute their results to the lower resistance to ion migration in micropores in CF, as assessed using a Nyquist impedance plot. However, the comparison was disturbed by the different origins of the ACs: graphitizable PAN and non-graphitizable phenol-formaldehyde resin.

**Figure 23:**
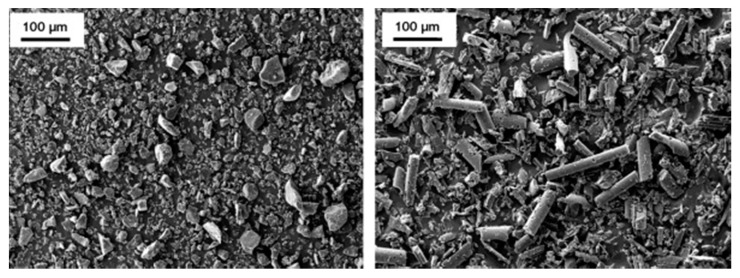
SEM images of activated particulate activated carbon (**left**) and pulverized activated carbon fibers (**right**). Adapted from [179].

More valuable results were presented by Torchala et al. [179]. In their study, particulate and fibrous ACs were derived from the same graphitizable precursor: synthetic anthracene oil-based pitch. The porosity was developed by CO_2_ activation at 900 °C until 50 wt% burn-off was achieved. The morphologies of the materials are shown in Figure 23. The PAC-type material had a wide particle size distribution (from a few to 100 μm). The pulverization of ACF resulted in the partial destruction of the fibrous structure into fine particles with a diameter of ∼20 μm.

The materials have similar BET surface areas of 1400–1450 m^2^ g^−1^ and L_0_ values of 1.00–1.09 nm. The same raw material and treatment conditions yielded comparable carbon structure ordering and surface oxygen contents (2.5–3.3 wt%).

**Figure 24:**
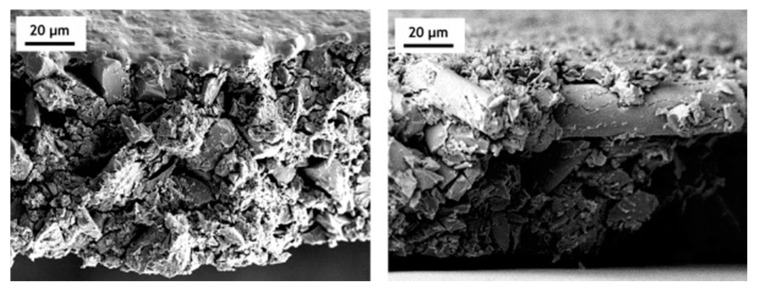
SEM images of cross-section of PAC-based (**left**) and ACF-based (**right**) working electrodes. Adapted from [179].

The morphologies of the ACs were preserved after pressing with the binder to form working electrodes (Figure 24). The fine particles were oriented mainly perpendicularly to the pressing force. Unexpectedly, the ACF-based electrodes had a higher packing density than the PAC-based ones (0.75 and 0.64 g cm^−3^, respectively).

**Figure 25:**
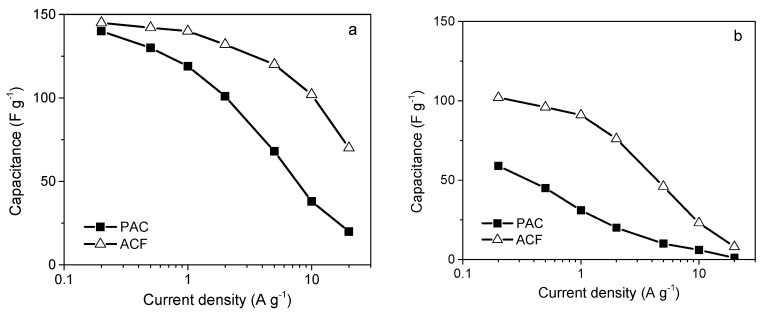
Rate capability of powdered activated carbon (PAC) and pulverized activated carbon fibres (ACF) in 6 mol L^−1^ KOH (**a**) and 0.5 mol L^−1^ K_2_SO_4_ (**b**). Adapted from [179].

The electrochemical behavior was assessed in 6 mol L^−1^ KOH and 0.5-M K_2_SO_4_ aqueous electrolytes. In both media, the fibrous form of the active material facilitated strong improvements in the capacitor performance. However, the effect was more pronounced at an enhanced current load or in the presence of lower-mobility sulfate anions (Figure 25). 

The rapid decrease in the specific surface capacity of the pulverized ACF at a high current density in 0.5 mol L^−1^ K_2_SO_4_ can be explained by the lower conductivity of the electrolyte. All of these results suggest better accessibility of the ACF porous system to ions. This accessibility is, in turn, related to the characteristic micropores, which are wedge shaped and exposed directly to the particle exteriors.

### 4.4. The Impurities in Activated Carbons

The purity of ACs is a very important parameter that influences the performance of the resulting supercapacitors. Problems with purity are commonly related to the presence of mineral matter, but in the case of organic electrolytes, adsorbed water and surface functionalities are also classified as “impurities”. 

Because of the oxidative conditions used in the activation process, the resultant carbon is rich in oxygen functionalities. The oxygen content depends on the activation method and process temperature and can vary over a wide range of 3–20 wt%. The surfaces of ACs should be “cleaned” by heat treatment in an inert or reductive atmosphere before application in EDLCs operating in organic electrolytes. Residual oxygen can cause irreversible redox reactions with the electrolyte, resulting in the release of gaseous products [182,183,184,185]. In the first stage, the evolved gases block the pores of the AC and separator, drastically decreasing the major cell parameters, such as capacity [186,187,188], self-discharge [189] and the usable voltage window [190]. In the longer term, excessive pressure inside the cell may damage the device and result in the leakage of a toxic and flammable electrolyte [191,192].

The surface functionalities do not have to be completely removed if the AC is utilized exclusively for aqueous-based EDLCs. Indeed, substantial research effort has been aimed at enhancing the oxygen content of the parent AC. The presence of oxygen is advantageous because it increases the overall capacity via the additional redox reactions between the AC surface and ions. This phenomenon is called “pseudocapacity”. However, several serious drawbacks can be observed when an AC with an extremely highly oxidized surface is applied in an aqueous supercapacitor. An excess of oxygen functionalities on the carbon surface decreases the inter-particle electric conductivity of the electrode material. This effect is more pronounced when the discharge rate of the supercapacitor is increased.

**Figure 26:**
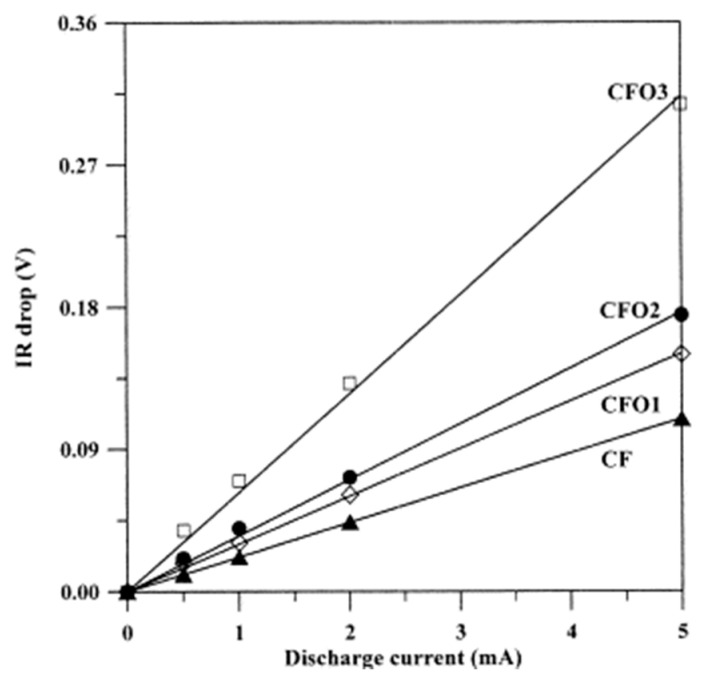
Variation of IR drop with discharge current for the parent (CF) and oxidized (CFOx) activated carbon fibers tested in 1 mol L^−1^ H_2_SO_4_ as an electrolyte. Reproduced from [174] with permission from Elsevier © 2002.

The negative influence of surface functionalities on the electrode resistance was reported by Hsieh et al. [174]. These authors oxidized PAN-based AC fabrics in gaseous oxygen at 250 °C for 0.5–6 h. The resultant materials were galvanostatically charged/discharged in an electrolyte of 1 mol L^−1^ H_2_SO_4_. The oxygen built into the fibers was determined by XPS and temperature-programmed desorption (TPD). An increase in oxygen concentration on the carbon surface of approximately 36% (treatment for 0.5 h) resulted in an equal increase in the discharge ohmic drop, whereas when the oxygen concentration was more than doubled (treatment for 6 h), the cell resistance increased by 260% (Figure 26). 

**Figure 27:**
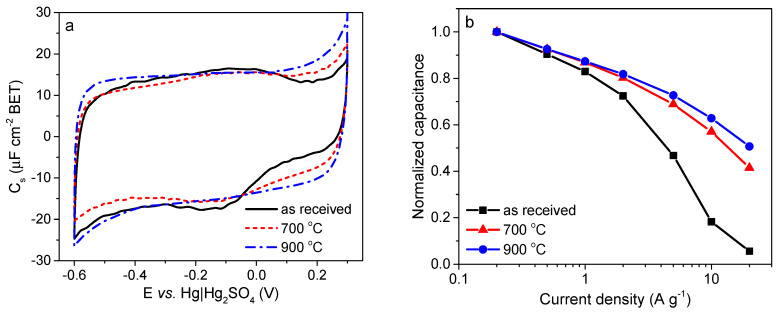
CV curves (**a**) and rate capability (**b**) of the as received H_3_PO_4_ activated carbon and the products of heat-treatment at 700 and 900 °C in nitrogen. The properties were evaluated in a 3-electrode system in 1 mol L^−1^ H_2_SO_4_ as an electrolyte. CV scan rate: 2 mV s^−1^.

The surfaces of chemically activated ACs are usually very rich in oxygen functionalities. Applying such materials as electrodes in EDCLs is inadvisable. The heat treatment of ACs in an inert (nitrogen or argon) or reductive (hydrogen) atmosphere is commonly performed to control the oxygen content. The electrochemical behaviors of a series of H_3_PO_4_ ACs after treatment at different temperatures are shown in Figure 27. This activation method was chosen intentionally because the low temperature of the process (480 °C) results in a relatively high oxygen content (17.5 wt%) in the parent AC. Moreover, the resulting ACs show a mixed micro-/mesoporous texture, which ensures good ion mobility. The thermal decomposition of oxygen groups was followed by a decrease in the BET surface area from 1370 to 1210 m^2^ g^−1^. Hence, to better observe the effect of oxygen, the electrochemical capacities were normalized to the SSA. 

At both annealing temperatures (700 and 900 °C), the oxygen content was reduced to a similar value of approximately 10.5 wt%, as indicated by the reduction of the pseudocapacitive humps in the CV curves at a scan rate of 2 mV s^−1^ (Figure 27a). However, under these charge/discharge conditions, the specific surface capacity (Cs) related to the EDL seems to increase slightly as the oxygen is removed. As both heat-treated materials have similar oxygen contents, they show comparable behaviors when discharged at a higher current load (Figure 27b). The relative capacity of the parent AC decreases to nearly zero at 20 A g^−1^, whereas the two heat-treated materials retain more than 40% of their initial capacitance values. This phenomenon can be explained by the improved electric conductivity of the AC resulting from surface oxygen removal and enhanced carbon structure ordering. As non-graphitizable wood was used as the raw material for activation, we believe that the effect of oxygen is dominant in this case.

The problem of low inter-particle electric conductivity of the electrode material mainly concerns chemically activated carbons (H_3_PO_4_ and KOH activation). This process is usually carried out at a lower temperature than physical activation and the formation of numerous surface oxygen groups is thermodynamically favored. However, this phenomenon should not affect the choice of the activation method. As it has been proven above, excess oxygen in AC can be easily reduced by simple and scalable thermal treatment in an inert atmosphere. The carboxylic groups are eliminated first as they show the least thermal stability. This is a beneficial phenomenon because a high contribution of carboxylic moieties or other acidic groups is particularly harmful. In addition to their influence on internal cell resistance, the presence of these functionalities on the carbon surface noticeably narrows the usable voltage window of the capacitor [193]. Moreover, the gradual decomposition of these species during cycling results in the release of a mixture of CO_2_ and CO within the cell [194], which leads to a gradual increase in the internal resistance and a decrease in the nominal capacity of the device. 

Mineral matter is also an important issue in both water- and organic-based supercapacitors. This matter acts as a ballast, decreasing the overall gravimetric capacity of the AC. Additionally, it causes a parasitic charge shuttle between the electrode and an electrolyte. The AC precursor is the major source of mineral matter in the final product. Hence, pure synthetic or selected lignocellulosic raw materials are preferred for the manufacturing of ACs for application in EDLCs. The direct use of coal is not recommended.

The mineral matter of an organic precursor accumulates in the resultant AC when steam or CO_2_ activation is applied. The effect of chemical activation is controversial. Selected parent minerals, such as silica and heavy metals, are removed during activation, but new inorganic species derived from the activation agent are introduced into the final carbon [195]. Thorough washing of chemically activated (or self-activated) materials with strong acids (generally HCl or HF) is commonly performed to remove all residual alkali metals, such as K, Na, Ca and Mg [126,128]. Unfortunately, the effect of AC purification on capacitor performance has not been reported. Many authors performing basic research on supercapacitors ignore the presence of mineral matter in ACs. However, some valuable information can be found in industrial reports focused on devices operating with organic electrolytes. In such media, heavy or alkali metals can be easily extracted from the positive electrode and deposited on the negative electrode in the form of dendrites. Initially, the presence of contaminants increases the self-discharge of the cell, but in the long term, the growth of the dendrites promotes internal short circuits and device failure. Therefore, the contents of heavy and alkali metals in ACs should be restricted to 20–50 ppm and 100 ppm, respectively [196,197]. 

**Figure 28:**
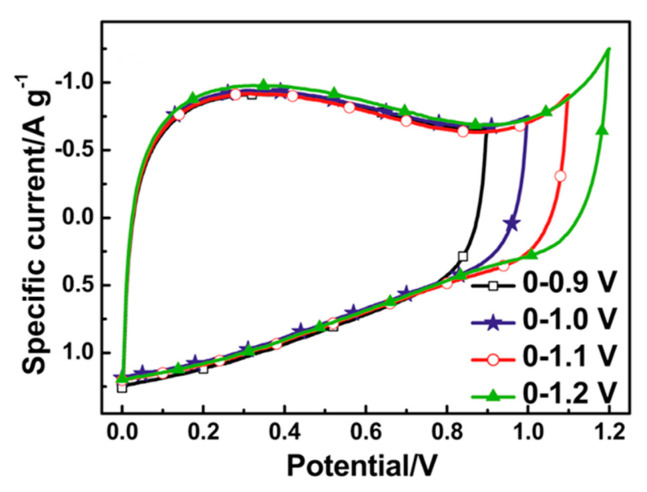
CV curves of the symmetric 2-electrode cell build with H_3_PO_4_ activated carbon and charged to 0.9, 1.0, 1.1 and 1.2 V in 6 mol L^−1^ KOH. Reproduced from [198] with permission from Elsevier © 2015.

In contrast, several researchers have proven that phosphorus retained on the surface of H_3_PO_4_ ACs can positively influence their electrochemical behavior in aqueous electrolytes. Qu et al. [198] reported that less than 2 at.% of phosphorus on the carbon surface facilitates over-charging a symmetric cell in 6 mol L^−1^ KOH above 1.0 V without significant water decomposition (Figure 28).

More spectacular results were presented by Huang and Hulicova-Jurcakova [74,199,200]. These authors found that H_3_PO_4_-activated lignocellulosic material with 2.6 at.% P can operate stably in a H_2_SO_4_-based symmetric capacitor at a cell voltage of 1.5 V, which is almost 0.3 V above the thermodynamic limit for aqueous electrolytes. The wide operating voltage window was explained by the anti-oxidative properties of P-containing carbon (positive electrode) and by the over-potential on the negative electrode related to reversible water-derived hydrogen storage.

## 5. Summary

This review presents an overview of the manufacture of ACs as electrode materials for EDLCs. The experimental data show that the design of a high-performance EDLC device should begin as early as the selection of the precursor for the porous electrode material. The price and availability of the precursor should not be the deciding factors for choosing the raw material. Indeed, the type of precursor chosen determines the subsequent activation method. Lignocellulosic materials are preferentially subjected to H_3_PO_4_ and ZnCl_2_ activation. In contrast, chemical activation using KOH/NaOH and conventional steam and CO_2_ gasification seem to be more universal and can be applied to produce ACs from various natural and synthetic materials. If possible, a graphitizable material with a good capacity to form a well-ordered carbon structure is recommended for activation because such materials show higher intrinsic conductivity and ensure more efficient EDL formation on the surface of the resultant AC. This holds true regardless of the electrolyte chemistry of EDLC cell. Chemical activation is commonly indicated as the most disturbing process for the structure ordering of a raw material, since this type of activation is usually carried out in too drastic conditions, which leads to the overdevelopment of porosity. However, while maintaining the similar porosity of the final AC, there is no direct correlation between the activation method and disordering of its structure.

CO_2_, steam and KOH activation can be applied to produce carbons with narrow porosities, whereas H_3_PO_4_ can be used to obtain ACs with high contributions of mesopores. However, the presented data indicate that this principle cannot be generalized. By manipulating variables such as the precursor origin, pretreatment temperature, process temperature and catalytic metal addition, we can produce mesoporous or wide-microporous carbons via physical and KOH activation. Similarly, ACs with high contributions of micropores can be obtained by H_3_PO_4_ activation.

KOH activation is the most commonly used laboratory method for producing ACs for use in EDLCs because of its ability to develop extremely high porosity coupled with reasonable process yields. However, the resultant porosity measured by gas adsorption is not always directly related to the EDLC performance. It should be emphasized that in most applications, the volumetric capacity is more important than the gravimetric capacity. Hence, the development of carbon porosity above 2000 m^2^ g^−1^ is unjustified. In this case, physical activation can be used, and its low cost and simplicity make this process important, especially on an industrial scale. 

Looking at the principle of EDLC operation, the specific surface area of electrode material should be the most important parameter. However, many studies have demonstrated that the optimal pore size distribution of an AC is no less important than its surface area. The pore size should be larger than the expected ion dimensions, bearing in mind the solvation phenomenon. Moreover, a certain contribution of wider pores is desired to ensure fast ion transfer.

Carbon purity is often ignored in basic research. However, it is a key factor for EDLCs with long cycle lives. The heavy metal content is recommended to be minimized in all manufacturing steps, especially in the precursor selection and post-activation purification steps. The amount of surface functionalities should also be restricted. The advantages of the pseudocapacitance effect and wider operating voltage window (related to the oxidative over-potential) should be not wasted because of the effects of low inter-particle conductivity and chemical surface stability.

Notwithstanding the general advices above, selection of the AC precursor and the activation method must be always performed individually, depending on the type of electrolyte used in EDLC cell. Physical activation of lignocellulosic raw materials is the most common process of the production of AC working in an organic electrolyte. In this case, it is easy to achieve high purity of the electrode material. Limited porosity is not a crucial problem here because in this system the energy density of the capacitor is more dependent on its operating voltage than on the specific capacity of AC. Chemical activation should be ideal for AC production for low-voltage EDLC with an aqueous electrolyte. A wide range of raw materials used, the ability to develop strong microporosity and create numerous surface functionalities (pseudo-capacity) are the most important advantages of this activation method. However, the economic aspect is still a barrier to the widespread use of chemically activated carbons in EDLC manufacturing.

## Figures and Tables

**Table 1:** Biopolymer composition of selected lignocellulosic materials used for AC preparation (wt%, dry basis).

Raw Material	Cellulose	Lignin	Hemicellulose	Ref.
Oak wood	39	28	19	[4]
Birch wood	42	20	21	[4]
Coconut shell *	40	32	28	[5]
Palm kernel shell	33	46	24	**
Almond shell *	51	20	29	[5]
Hazelnut shell *	27	43	30	[5]
Groundnut shell	36	30	19	[6]
Rice husk	31	14	24	[6]
Bagasse	41	18	23	[6]
Coir pith	29	31	15	[6]
Olive stones	32	27	22	[7]
Peach stones	46	33	14	[8]
Hemp stem	51	13	20	[9]
Sugarcane bagasse	32–44	19–24	27–32	[10]
Wheat straw	33–40	15–20	20–25	[11]
Softwood	35–40	27–30	25–30	[11]
Hardwood	45–50	20–25	20–25	[11]

* Dry ash free basis, ** our work.

**Table 2:** Char yields obtained on carbonization of various lignocellulosic, polymeric and carbonaceous raw materials.

Raw Material	Temperature °C	Char Yield wt%
Cellulose	800	19
Lignin	800	43
Palm kernel shell	900	33
Walnut shells	800	26
Coconut shells	800	29
Hazelnut shells	800	25
Cherry stones	800	25
Chitozan	800	30
PFA	800	47
PET	800	19
PAN	550	44
PANox	550	67
Polyvinylpyridine (PVP)	550	9
PVPox	550	60
Phenol formaldehyde resin	800	43
Coal-tar pitch	800	45
Lignite	800	45–55
High-volatile bituminous coal	800	60–70

**Table 3:** Porosity parameters of semi-cokes obtained from pyrolysis of bituminous coal and a mixture of coal/KOH at the ratio of 1:4. Temperature ramp: 5° min^−1^. Final temperature: 520 °C. Soaking time 2 h. Argon as a purge gas.

Conditions	Burn-Off (wt%)	S_BET_ (m^2^ g^−1^)	V_T_ (cm^3^ g^−1^)	V_DR_ (cm^3^ g^−1^)	L_0_ (nm)
**KOH activation**	25.5	1710	0.73	0.59	1.41
**Pyrolysis**	21.5	0	0	n.d.	n.d.

**Table 4:** Porosity parameters of ACs produced from coal-tar mesophase by KOH activation (mesophase/KOH ratio 1:3, soaking 2 h).

Reaction Temperature (°C)	Burn-Off (wt%)	S_BET_ (m^2^ g^−1^)	V_T_ (cm^3^ g^−1^)	V_DR_ (cm^3^ g^−1^)	L_0_ (nm)	V_DR_/V_T_
**600**	21.6	1790	0.71	0.66	1.11	0.93
**650**	22.8	1960	0.76	0.71	1.18	0.93
**700**	22.7	2410	1.01	0.80	1.20	0.79
**750**	22.8	2500	1.04	0.82	1.21	0.79
**800**	27.5	2590	1.12	0.82	1.29	0.73
**850**	33.2	2710	1.18	0.84	1.36	0.71
**900**	45.3	2610	1.10	0.84	1.33	0.76

**Table 5:** Porosity parameters of ACs produced by KOH activation of bituminous coal-derived chars (char/KOH ratio 1:3, reaction temperature 800 °C, soaking 1 h).

Pre-Carbonization Temperature (°C)	Burn-Off (wt%)	S_BET_ (m^2^ g^−1^)	L_0_ (nm)	V_DR_/V_T_
**520**	34.5	3030	1.40	0.71
**600**	27.9	2550	1.34	0.82
**700**	24.9	2030	1.17	0.98
**800**	24.4	1550	0.94	0.91
**900**	26.2	1310	0.84	0.92
**1000**	18.6	990	0.79	0.92

**Table 6:** Porosity parameters of ACs produced by KOH activation of pitch-derived cokes (coke/KOH ratio 1:3, reaction temperature 800 °C, soaking 1 h).

Pre-Carbonization Temperature (°C)	Burn-Off (wt%)	S_BET_ (m^2^ g^−1^)	L_0_ (nm)	V_DR_/V_T_
**520**	22.4	2640	1.36	0.76
**600**	18.8	1450	1.23	0.74
**700**	23.7	1410	1.06	0.81
**800**	17.0	190	2.53	0.55
**900**	16.2	~0	n.d.	n.d.
**1000**	9.3	~0	n.d.	n.d.

**Table 7:** Summary of optimal experimental conditions in terms of porosity development of ACs produced by H_3_PO_4_ activation.

Precursor	H_3_PO_4_ g/g Precursor	Temp. (°C)	Soaking Time (min)	S_BET_ (m^2^ g^−1^)	V_mic_ (cm^3^ g^−1^)	V_mic/_V_T_	Ref.
Birch	3.1	480	20	1826	0.67	0.46	[4]
Oak	3.1	480	20	2553	0.94	0.59	[4]
Hemp stem	1.6	480	10	2215	0.859	0.52	[9]
Corncob	1	400	60	2071	0.78	0.69	[81]
Eucalyptus residue	2.5	400	180	1545	0.04	0.02	[87]
Cotton stalks	1.5	500	120	1720	0.71	0.80	[93]
Olive stone	1.3	400	120	1740	0.73	0.82	[94]
Rice hull	1.5	450	-	1295	0.46	0.63	[95]
Sugar cane bagasse	1.5	500	60	1132	0.61	0.54	[96]

**Table 8:** Summary of experimental conditions in terms of porosity development of ACs produced by ZnCl_2_ activation.

Precursor	g ZnCl_2_/g Precursor	Temp. (°C)	Soaking Time (min)	S_BET_ (m^2^ g^−1^)	V_mic_ (cm^3^ g^−1^)	V_mic/_V_T_	Ref.
Cherry stones	3	700	120	1704	0.98	0.63	[100]
Fox nut	2	600	60	2869	1.68	0.86	[101]
Cherry stones	3	500	120	1566	0.65	0.81	[104]
Macadamia nutshell	1	500	60	1718	0.72	0.88	[107]
Palm shell	1.35	500	120	1672	0.87	0.88	[112]
Chestnut shell	2	800	90	1824	0.87	0.89	[116]
Rice husk	2	500	60	2434	0.59	0.44	[121]
Sugar beet bagasse	3	700	90	1826	0.71	0.74	[122]
Waste coffee grounds	1	850	60	1019	0.34	0.71	[123]

**Table 9:** Effective size of selected ions and their solvation free energy (based on various reports).

Ion	Non-Solvated Ion Size (nm)	Solvated Ion Size (nm)	Solvation Free Energy (kJ mol^−1^)
Organic electrolyte			
TEA^+^	0.67–0.75	1.30 (in acetonitrile) 1.74 (in propylene carbonate)	
BF_4_^−^	0.48–0.50	1.16 (in acetonitrile) 1.54 (in propylene carbonate)	
TFSI^−^	0.37	1.35 (in ethylene carbonate)	
Water-based electrolyte			
Li^+^	0.15–0.18	0.68–0.76	515–544
Na^+^	0.20–0.23	0.55–0.72	365–435
K^+^	0.27–0.30	0.40–0.66	271–351
NH_4_^+^	0.30–0.32	0.66	
Mg^2+^	0.12–0.14	0.60–0.94	1828–1922
Ca^2+^	0.20–0.25	0.82–0.84	1306–1650
Cl^−^	0.33–0.39	0.65–0.66	340–371
I^−^	0.41–0.43	0.60	254
SO_4_^2−^	0.43–0.58	0.60–0.74	1145
NO_3_^−^	0.38	0.67–0.68	328
ClO_4_^−^	0.45–0.47	0.60	238

## Abbreviations

AC	Activated carbon
ACF	Activated carbon fiber
BET	Brunauer-Emmett-Teller
CEC	Cation exchange capacity
DFT	Density functional theory
DR	Dubinin-Radushkevich
EDL	Electric double layer
EDLC	Electric double layer capacitor
ESEM	Environmental scanning electron microscopy
FESEM	Field emission scanning electron microscopy
HRTEM	High-resolution transmission electron microscopy
HTT	Heat treatment temperature
IUPAC	International Union of Pure and Applied Chemistry
L_0_	Mean micropore size
L_a_	Lateral size of crystallite
L_c_	Height of crystallite
LiTFSI	Bis(trifluoromethane)sulfonamide lithium salt
MCMB	Mesocarbon microbeads
MWCNTs	Multi-wall carbon nanotubes
NLDFT	Non-local density functional theory
PAC	Powdered activated carbon
PAN	Polyacrylonitrile
PET	Poly(ethylene terephthalate)
PFA	Poly(furfuryl alcohol)
PVP	Polyvinylpyridine
SANS	Small-angle neutron scattering
SAXS	Small-angle X-ray scattering
SEM	Scanning electron microscopy
SQDFT	Quenched solid density functional theory
SSA	Specific surface area
TEABF_4_	Tetraethylammonium tetrafluoroborate
TPD	Temperature-programmed desorption
XPS	X-ray photoelectron spectroscopy

## References

[1] Abioye A.M., Ani F.N. (2015). Recent development in the production of activated carbon electrodes from agricultural waste biomass for supercapacitors: A review. Renew. Sustain. Energ. Rev..

[2] Kalyani P., Anitha A. (2013). Biomass carbon & its prospects in electrochemical energy systems. Int. J. Hydrog Energy.

[3] Dias J.M., Alvim-Ferraz M.C.M., Almeida M.F., Rivera-Utrilla J., Sánchez-Polo M. (2007). Waste materials for activated carbon preparation and its use in aqueous-phase treatment: A review. J. Environ. Manag..

[4] Klijanienko A., Lorenc-Grabowska E., Gryglewicz G. (2007). Development of mesoporosity during phosphoric acid activation of wood in steam atmosphere. Bioresour. Technol..

[5] Vassilev S.V., Baxter D., Andersen L.K., Vassileva C.G., Morgan T.J. (2012). An overview of the organic and inorganic phase composition of biomass. Fuel.

[6] Raveendran K., Ganesh A., Khilar K.C. (1995). Influence of mineral matter on biomass pyrolysis characteristics. Fuel.

[7] Rodríguez G., Lama A., Rodríguez R., Jiménez A., Guillén R., Fernández-Bolaños J. (2008). Olive stone an attractive source of bioactive and valuable compounds. Bioresour. Technol..

[8] Uysal T., Duman G., Onal Y., Yasa I., Yanik J. (2014). Production of activated carbon and fungicidal oil from peach stone by two-stage process. J. Anal. Appl. Pyrolysis.

[9] Lupul I., Yperman J., Carleer R., Gryglewicz G. (2015). Tailoring of porous texture of hemp stem-based activated carbon produced by phosphoric acid activation in steam atmosphere. J. Porous Mater..

[10] Saidur R., Abdelaziz E.A., Demirbas A., Hossain M.S., Mekhilef S. (2011). A review on biomass as a fuel for boilers. Renew. Sustain. Energ. Rev..

[11] McKendry P. (2002). Energy production from biomass (Part 1): Overview of biomass. Bioresour. Technol..

[12] Śliwak A., Díez N., Miniach E., Gryglewicz G. (2016). Nitrogen-containing chitosan-based carbon as an electrode material for high-performance supercapacitors. J. Appl. Electrochem..

[13] Yuan X., Lee J.G., Yun H., Deng S., Kim Y.J., Lee J.E., Kwak S.K., Lee K.B. (2020). Solving two environmental issues simultaneously: Waste polyethylene terephthalate plastic bottle-derived microporous carbons for capturing CO_2_. Chem. Eng. J..

[14] Castro C.S.D., Viau L.N., Andrade J.T., Mendonça T.A.P., Gonçalves M. (2018). Mesoporous activated carbon from polyethyleneterephthalate (PET) waste: Pollutant adsorption in aqueous solution. New J. Chem..

[15] Bratek W., Świątkowski A., Pakuła M., Biniak S., Bystrzejewski M., Szmigielski R. (2013). Characteristics of activated carbon prepared from waste PET by carbon dioxide activation. J. Anal. Appl. Pyrolysis.

[16] Nasr M.F., Mosleh S.E.-S., Ali S.S.M., Abo El-Ola S.M. (2019). Tailored removal of zinc and chrome ions by the adsorption onto nonwoven ACF (activated carbon fiber) prepared from PAN (polyacrylonitrile) waste. Desalin. Water Treat..

[17] Lorenc-Grabowska E., Gryglewicz G., Diez M.A. (2013). Kinetics and equilibrium study of phenol adsorption on nitrogen-enriched activated carbons. Fuel.

[18] Gonsalvesh L., Marinov S.P., Gryglewicz G., Carleer R., Yperman J. (2016). Preparation, characterization and application of polystyrene based activated carbons for Ni(II) removal from aqueous solution. Fuel Process. Technol..

[19] Hu W., Cheng S., Xia H., Zhang L., Jiang X., Zhang Q., Chen Q. (2019). Waste phenolic resin derived activated carbon by microwave-assisted KOH activation and application to dye wastewater treatment. Green Process. Synth..

[20] Choma J., Osuchowski Ł., Marszewski M., Jaroniec M. (2014). Highly microporous polymer-based carbons for CO_2_ and H_2_ adsorption. RSC Adv..

[21] Yue Z., Dunya H., Ashuri M., Kucuk K., Aryal S., Antonov S., Alabbad B., Segre C.U., Mandal B.K. (2020). Synthesis of a very high specific surface area active carbon and its electrical double-layer capacitor properties in organic electrolytes. ChemEngineering.

[22] Lorenc-Grabowska E., Diez M.A., Gryglewicz G. (2016). Influence of pore size distribution on the adsorption of phenol on PET-based activated carbons. J. Colloid Interface Sci..

[23] Grzyb B., Machnikowski J., Weber J.V. (2004). Mechanism of co-pyrolysis of coal-tar pitch with polyvinylpyridine. J. Anal. Appl. Pyrolysis.

[24] Carrott P.J.M., Ribeiro Carrott M.M.L., Correia P.F.M.M. (2018). Evolution of porosity of activated carbon fibres prepared from pre-oxidized acrylic fibres. Microporous Mesoporous Mater..

[25] Franklin R.E. (1951). Crystallite growth in graphitizing and non-graphitizing carbons. Proc. R. Soc. Lond. A.

[26] Marsh H., Walker P.L., Walker P.L., Thrower P.A. (1994). The Formation of Graphitizable Carbons via Mesophase: Chemical and Kinetic Considerations. Chemistry and Physics of Carbon.

[27] Stoeckli H.F. (1990). Microporous carbons and their characterization: The present state of the art. Carbon.

[28] Marsh H., Wynne-Jones W.F.K. (1964). The surface properties of carbon-I the effect of activated diffusion in the determination of surface area. Carbon.

[29] Shcherban N.D., Yaremov P.S., Ilyin V.G., Ovcharova M.V. (2014). Influence of the method of activation on the structural and sorption properties of the products of carbonization of sucrose. J. Anal. Appl. Pyrolysis.

[30] Lin S.-H., Hsu L.-Y., Chou C.-S., Jhang J.-W., Wu P. (2014). Carbonization process of Moso bamboo (Phyllostachys pubescens) charcoal and its governing thermodynamics. J. Anal. Appl. Pyrolysis.

[31] Rouzaud J.-N., Clinard C. (2002). Quantitative high-resolution transmission electron microscopy: A promising tool for carbon materials characterization. Fuel Process. Technol..

[32] Rodríguez-Reinoso F., Molina-Sabio M. (1998). Textural and chemical characterization of microporous carbons. Adv. Colloid Interface Sci..

[33] Shi H. (1996). Activated carbons and double layer capacitance. Electrochim. Acta.

[34] Neimark A.V., Lin Y., Ravikovitch P.I., Thommes M. (2009). Quenched solid density functional theory and pore size analysis of micro-mesoporous carbons. Carbon.

[35] Bardestani R., Patience G.S., Kaliaguine S. (2019). Experimental methods in chemical engineering: Specific surface area and pore size distribution measurements—BET, BJH, and DFT. Can. J. Chem. Eng..

[36] Jagiello J., Thommes M. (2004). Comparison of DFT characterization methods based on N_2_, Ar, CO_2_, and H_2_ adsorption applied to carbons with various pore size distributions. Carbon.

[37] Stoeckli F., López-Ramón M.V., Hugi-Cleary D., Guillot A. (2001). Micropore sizes in activated carbons determined from the Dubinin–Radushkevich equation. Carbon.

[38] Denoyel R., Fernandez-Colinas J., Grillet Y., Rouquerol J. (1993). Assessment of the surface area and microporosity of activated charcoals from immersion calorimetry and nitrogen adsorption data. Langmuir.

[39] Stoeckli F., Hugi-Cleary D. (2001). On the mechanisms of phenol adsorption by carbons. Russ. Chem. Bull..

[40] Stoeckli H.F., Kraehenbuehl F. (1981). The enthalpies of immersion of active carbons, in relation to the Dubinin theory for the volume filling of micropores. Carbon.

[41] Ruike M., Kasu T., Setoyama N., Suzuki T., Kaneko K. (1994). Inaccessible pore characterization of less-crystalline microporous solids. J. Phys. Chem..

[42] Kierzek K., Machnikowski J. (2018). Cellulose-derived carbons as a high performance anodic material for Na-ion battery. Ionics.

[43] Hoinkis E., Thrower P.A. (1997). Small Angle Scattering of Neutrons and X-rays from Carbons and Graphites. Chemistry and Physics of Carbon.

[44] Nishikawa K., Yusada E., Inagaki M., Kaneko K., Endo M., Oya A., Tanabe Y. (2003). Pore Structure Analyses of Carbons by Small-Angle X-ray Scattering. Carbon Alloys: Novel Concepts to Develop Carbon Science and Technology.

[45] Li Y., Lu Y., Meng Q., Jensen A.C.S., Zhang Q., Zhang Q., Tong Y., Qi Y., Gu L., Titirici M.-M. (2019). Regulating pore structure of hierarchical porous waste cork-derived hard carbon anode for enhanced na storage performance. Adv. Energy Mater..

[46] Calo J.M., Hall P.J., Antxustegi M. (2001). Carbon porosity characterization via small angle neutron scattering. Colloids Surf. A Physicochem. Eng..

[47] Sakurovs R., Koval L., Grigore M., Sokolova A., de Campo L., Rehm C. (2018). Nanostructure of cokes. Int. J. Coal Geol..

[48] Biniak S., Świątkowski A., Pakuła M., Radovic L.R. (2001). Electrochemical Studies of Phenomena at Active Carbon-Electrolyte Solutions Interfaces. Chemistry and Physics of Carbon.

[49] Guan T., Zhao J., Zhang G., Zhang D., Han B., Tang N., Wang J., Li K. (2018). Insight into controllability and predictability of pore structures in pitch-based activated carbons. Microporous Mesoporous Mater..

[50] Rodríguez-Reinoso F., Molina-Sabio M., González M.T. (1995). The use of steam and CO_2_ as activating agents in the preparation of activated carbons. Carbon.

[51] Molina-Sabio M., Gonzalez M.T., Rodriguez-Reinoso F., Sepúlveda-Escribano A. (1996). Effect of steam and carbon dioxide activation in the micropore size distribution of activated carbon. Carbon.

[52] Lillo-Ródenas M.A., Lozano-Castelló D., Cazorla-Amorós D., Linares-Solano A. (2001). Preparation of activated carbons from Spanish anthracite. Carbon.

[53] Lillo-Ródenas M.A., Cazorla-Amorós D., Linares-Solano A. (2003). Understanding chemical reactions between carbons and NaOH and KOH. Carbon.

[54] Maciá-Agulló J.A., Moore B.C., Cazorla-Amorós D., Linares-Solano A. (2004). Activation of coal tar pitch carbon fibres: Physical activation vs. chemical activation. Carbon.

[55] Linares-Solano A., Lozano-Castello D., Lillo-Ródenas M.A., Cazorla-Amorós D., Radovic L.R. (2008). Carbon Activation by Alkaline Hydroxides Preparation and Reactions, Porosity and Performance. Chemistry and Physics of Carbon.

[56] Kierzek K., Frackowiak E., Lota G., Gryglewicz G., Machnikowski J. (2004). Electrochemical capacitors based on highly porous carbons prepared by KOH activation. Electrochim. Acta.

[57] Lillo-Ródenas M.A., Juan-Juan J., Cazorla-Amorós D., Linares-Solano A. (2004). About reactions occurring during chemical activation with hydroxides. Carbon.

[58] Lozano-Castelló D., Cazorla-Amorós D., Linares-Solano A., Quinn D.F. (2002). Influence of pore size distribution on methane storage at relatively low pressure: Preparation of activated carbon with optimum pore size. Carbon.

[59] Xing B.-L., Guo H., Chen L.-J., Chen Z.-F., Zhang C.-X., Huang G.-X., Xie W., Yu J.-L. (2015). Lignite-derived high surface area mesoporous activated carbons for electrochemical capacitors. Fuel Process. Technol..

[60] Tellez-Juárez M.C., Fierro V., Zhao W., Fernández-Huerta N., Izquierdo M.T., Reguera E., Celzard A. (2014). Hydrogen storage in activated carbons produced from coals of different ranks: Effect of oxygen content. Int. J. Hydrog. Energy.

[61] Lozano-Castelló D., Cazorla-Amorós D., Linares-Solano A. (2002). Powdered activated carbons and activated carbon fibers for methane storage: A comparative study. Energy Fuels.

[62] Cheng G., Zhang C.X., Zhang X.M., Zhang Z.L. (2019). Preparation of lignite-based activated carbon and its flue gas desulfurization performance. Mater. Res. Express.

[63] Deng J., Peng Z., Xiao Z., Song S., Dai H., Li L. (2020). Porous doped carbons from anthracite for high-performance supercapacitors. Appl. Sci..

[64] Alkathiri D.S.S., Sabri M.A., Ibrahim T.H., ElSayed Y.A., Jumean F. (2020). Development of activated carbon fibers for removal of organic contaminants. Int. J. Environ. Sci. Technol..

[65] Raymundo-Piñero E., Cazorla-Amorós D., Linares-Solano A., Delpeux S., Frackowiak E., Szostak K., Béguin F. (2002). High surface area carbon nanotubes prepared by chemical activation. Carbon.

[66] Raymundo-Piñero E., Azaïs P., Cacciaguerra T., Cazorla-Amorós D., Linares-Solano A., Béguin F. (2005). KOH and NaOH activation mechanisms of multiwalled carbon nanotubes with different structural organisation. Carbon.

[67] Lozano-Castelló D., Lillo-Ródenas M.A., Cazorla-Amorós D., Linares-Solano A. (2001). Preparation of activated carbons from Spanish anthracite. Carbon.

[68] Di Blasi C., Galgano A., Branca C. (2009). Effects of potassium hydroxide impregnation on wood pyrolysis. Energy Fuels.

[69] Jagtoyen M., Derbyshire F. (1993). Some considerations of the origins of porosity in carbons from chemically activated wood. Carbon.

[70] Suárez-García F., Martínez-Alonso A., Tascón J.M.D. (2004). Activated carbon fibers from Nomex by chemical activation with phosphoric acid. Carbon.

[71] Castro-Muñiz A., Suárez-García F., Martínez-Alonso A., Tascón J.M.D. (2011). Activated carbon fibers with a high content of surface functional groups by phosphoric acid activation of PPTA. J. Colloid Interface Sci..

[72] Jagtoyen M., Derbyshire F. (1998). Activated carbons from yellow poplar and white oak by H_3_PO_4_ activation. Carbon.

[73] Solum M.S., Pugmire R.J., Jagtoyen M., Derbyshire F. (1995). Evolution of carbon structure in chemically activated wood. Carbon.

[74] Huang C., Puziy A.M., Sun T., Poddubnaya O.I., Suárez-García F., Tascón J.M.D., Hulicova-Jurcakova D. (2014). Capacitive behaviours of phosphorus-rich carbons derived from lignocelluloses. Electrochim. Acta.

[75] Puziy A.M., Poddubnaya O.I., Socha R.P., Gurgul J., Wisniewski M. (2008). XPS and NMR studies of phosphoric acid activated carbons. Carbon.

[76] Benaddi H., Bandosz T.J., Jagiello J., Schwarz J.A., Rouzaud J.N., Legras D., Béguin F. (2000). Surface functionality and porosity of activated carbons obtained from chemical activation of wood. Carbon.

[77] Puziy A.M., Poddubnaya O.I., Martínez-Alonso A., Castro-Muñiz A., Suárez-García F., Tascón J.M.D. (2007). Oxygen and phosphorus enriched carbons from lignocellulosic material. Carbon.

[78] Puziy A.M., Poddubnaya O.I., Martínez-Alonso A., Suárez-García F., Tascón J.M.D. (2005). Surface chemistry of phosphorus-containing carbons of lignocellulosic origin. Carbon.

[79] Suárez-García F., Villar-Rodil S., Blanco C.G., Martínez-Alonso A., Tascón J.M.D. (2004). Effect of phosphoric acid on chemical transformations during Nomex pyrolysis. Chem. Mater..

[80] Imamura R., Matsui K., Takeda S., Ozaki J., Oya A. (1999). A new role for phosphorus in graphitization of phenolic resin. Carbon.

[81] Sych N.V., Trofymenko S.I., Poddubnaya O.I., Tsyba M.M., Sapsay V.I., Klymchuk D.O., Puziy A.M. (2012). Porous structure and surface chemistry of phosphoric acid activated carbon from corncob. Appl. Surf. Sci..

[82] Molina-Sabio M., Rodríguez-Reinoso F., Caturla F., Sellés M.J. (1995). Porosity in granular carbons activated with phosphoric acid. Carbon.

[83] Molina-Sabio M., Rodríguez-Reinoso F. (2004). Role of chemical activation in the development of carbon porosity. Colloids Surf. A Physicochem. Eng..

[84] Girgis B.S., Ishak M.F. (1999). Activated carbon from cotton stalks by impregnation with phosphoric acid. Mater. Lett..

[85] Guo Y., Rockstraw D.A. (2007). Physicochemical properties of carbons prepared from pecan shell by phosphoric acid activation. Bioresour. Technol..

[86] Macías-García A., Carrasco-Amador J.P., Encinas-Sánchez V., Díaz-Díez M.A., Torrejón-Martín D. (2019). Preparation of activated carbon from kenaf by activation with H_3_PO_4_. Kinetic study of the adsorption/electroadsorption using a system of supports designed in 3D, for environmental applications. J. Environ. Chem. Eng..

[87] Han Q., Wang J., Goodman B.A., Xie J., Liu Z. (2020). High adsorption of methylene blue by activated carbon prepared from phosphoric acid treated eucalyptus residue. Powder Technol..

[88] Guo Y., Rockstraw D.A. (2006). Physical and chemical properties of carbons synthesized from xylan, cellulose, and Kraft lignin by H_3_PO_4_ activation. Carbon.

[89] Fierro V., Torné-Fernández V., Celzard A. (2006). Kraft lignin as a precursor for microporous activated carbons prepared by impregnation with ortho-phosphoric acid: Synthesis and textural characterization. Microporous Mesoporous Mater..

[90] Benaddi H., Legras D., Rouzaud J.N., Beguin F. (1998). Influence of the atmosphere in the chemical activation of wood by phosphoric acid. Carbon.

[91] Danish M., Ahmad T., Hashim R., Said N., Akhtar M.N., Mohamad-Saleh J., Sulaiman O. (2018). Comparison of surface properties of wood biomass activated carbons and their application against rhodamine B and methylene blue dye. Surf. Interfaces.

[92] Zhang Z., Lei Y., Li D., Zhao J., Wang Y., Zhou G., Yan C., He Q. (2020). Sudden heating of H_3_PO_4_-loaded coconut shell in CO_2_ flow to produce super activated carbon and its application for benzene adsorption. Renew. Energy.

[93] Nahil M.A., Williams P.T. (2012). Pore characteristics of activated carbons from the phosphoric acid chemical activation of cotton stalks. Biomass Bioenergy.

[94] Yavuz R., Akyildiz H., Karatepe N., Çetinkaya E. (2010). Influence of preparation conditions on porous structures of olive stone activated by H_3_PO_4_. Fuel Process. Technol..

[95] Guo Y., Rockstraw D.A. (2007). Activated carbons prepared from rice hull by one-step phosphoric acid activation. Microporous Mesoporous Mater..

[96] Castro J.B., Bonelli P.R., Cerrella E.G., Cukierman A.L. (2000). Phosphoric acid activation of agricultural residues and bagasse from sugar cane: Influence of the experimental conditions on adsorption characteristics of activated carbons. Ind. Eng. Chem. Res..

[97] Caturla F., Molina-Sabio M., Rodríguez-Reinoso F. (1991). Preparation of activated carbon by chemical activation with ZnCl_2_. Carbon.

[98] Marsh H., Rodriguez-Reinoso F. (2006). Activated Carbon.

[99] Kante K., Nieto-Delgado C., Rangel-Mendez J.R., Bandosz T.J. (2012). Spent coffee-based activated carbon: Specific surface features and their importance for H_2_S separation process. J. Hazard. Mater..

[100] Angin D. (2014). Production and characterization of activated carbon from sour cherry stones by zinc chloride. Fuel.

[101] Kumar A., Mohan Jena H. (2015). High surface area microporous activated carbons prepared from Fox nut (Euryale ferox) shell by zinc chloride activation. Appl. Surf. Sci..

[102] Ozdemir I., Şahin M., Orhan R., Erdem M. (2014). Preparation and characterization of activated carbon from grape stalk by zinc chloride activation. Fuel Process. Technol..

[103] Sun Y., Webley P.A. (2010). Preparation of activated carbons from corncob with large specific surface area by a variety of chemical activators and their application in gas storage. Chem. Eng. J..

[104] Olivares-Marín M., Fernández-González C., Macías-García A., Gómez-Serrano V. (2006). Preparation of activated carbon from cherry stones by chemical activation with ZnCl_2_. Appl. Surf. Sci..

[105] Abatan O.G., Oni B.A., Agboola O., Efevbokhan V., Abiodun O.O. (2019). Production of activated carbon from African star apple seed husks, oil seed and whole seed for wastewater treatment. J. Clean. Prod..

[106] Shrestha L.K., Shrestha R.G., Maji S., Pokharel B.P., Rajbhandari R., Shrestha R.L., Pradhananga R.R., Hill J.P., Ariga K. (2020). High surface area nanoporous graphitic carbon materials derived from lapsi seed with enhanced supercapacitance. Nanomaterials.

[107] Ahmadpour A., Do D.D. (1997). The preparation of activated carbon from macadamia nutshell by chemical activation. Carbon.

[108] Torregrosa R., Martín-Martínez J. (1991). Activation of lignocellulosic materials: A comparison between chemical, physical and combined activation in terms of porous texture. Fuel.

[109] Üner O., Geçgel Ü., Bayrak Y. (2019). Preparation and characterization of mesoporous activated carbons from waste watermelon rind by using the chemical activation method with zinc chloride. Arab. J. Chem..

[110] Nasrullah A., Saad B., Bhat A.H., Khan A.S., Danish M., Isa M.H., Naeem A. (2019). Mangosteen peel waste as a sustainable precursor for high surface area mesoporous activated carbon: Characterization and application for methylene blue removal. J. Clean. Prod..

[111] Spagnoli A.A., Giannakoudakis D.A., Bashkova S. (2017). Adsorption of methylene blue on cashew nut shell based carbons activated with zinc chloride: The role of surface and structural parameters. J. Mol. Liq..

[112] Hoseinzadeh Hesas R., Arami-Niya A., Wan Daud W.M.A., Sahu J.N. (2013). Comparison of oil palm shell-based activated carbons produced by microwave and conventional heating methods using zinc chloride activation. J. Anal. Appl. Pyrolysis.

[113] He X., Ling P., Qiu J., Yu M., Zhang X., Yu C., Zheng M. (2013). Efficient preparation of biomass-based mesoporous carbons for supercapacitors with both high energy density and high power density. J. Power Sources.

[114] Rufford T.E., Hulicova-Jurcakova D., Khosla K., Zhu Z., Lu G.Q. (2010). Microstructure and electrochemical double-layer capacitance of carbon electrodes prepared by zinc chloride activation of sugar cane bagasse. J. Power Sources.

[115] Liu Y., Wang Y., Zhang G., Liu W., Wang D., Dong Y. (2016). Preparation of activated carbon from willow leaves and evaluation in electric double-layer capacitors. Mater. Lett..

[116] Cheng L., Guo P., Wang R., Ming L., Leng F., Li H., Zhao X.S. (2014). Electrocapacitive properties of supercapacitors based on hierarchical porous carbons from chestnut shell. Colloids Surf. A Physicochem. Eng..

[117] Timur S., Kantarli I.C., Onenc S., Yanik J. (2010). Characterization and application of activated carbon produced from oak cups pulp. J. Anal. Appl. Pyrolysis.

[118] Lin H., Liu Y., Chang Z., Yan S., Liu S., Han S. (2020). A new method of synthesizing hemicellulose-derived porous activated carbon for high-performance supercapacitors. Microp. Mesop. Mat..

[119] Ramirez-Castro C., Schütter C., Passerini S., Balducci A. (2016). Microporous carbonaceous materials prepared from biowaste for supercapacitor application. Electrochim. Acta.

[120] Rawal S., Joshi B., Kumar Y. (2018). Synthesis and characterization of activated carbon from the biomass of Saccharum bengalense for electrochemical supercapacitors. J. Energy Storage.

[121] He X., Ling P., Yu M., Wang X., Zhang X., Zheng M. (2013). Rice husk-derived porous carbons with high capacitance by ZnCl_2_ activation for supercapacitors. Electrochim. Acta.

[122] Demiral H., Gündüzoğlu G. (2010). Removal of nitrate from aqueous solutions by activated carbon prepared from sugar beet bagasse. Bioresour. Technol..

[123] Rufford T.E., Hulicova-Jurcakova D., Fiset E., Zhu Z., Lu G.Q. (2009). Double-layer capacitance of waste coffee ground activated carbons in an organic electrolyte. Electrochem. Commun..

[124] Wu Y., Xia C., Cai L., Shi S.Q. (2018). Controlling pore size of activated carbon through self-activation process for removing contaminants of different molecular sizes. J. Colloid Interface Sci..

[125] Sun K., Leng C.-Y., Jiang J.-C., Bu Q., Lin G.-F., Lu X.-C., Zhu G.-Z. (2017). Microporous activated carbons from coconut shells produced by self-activation using the pyrolysis gases produced from them, that have an excellent electric double layer performance. New Carbon Mater..

[126] Raymundo-Piñero E., Cadek M., Béguin F. (2009). Tuning carbon materials for supercapacitors by direct pyrolysis of seaweeds. Adv. Funct. Mater..

[127] Raymundo-Piñero E., Leroux F., Béguin F. (2006). A high-performance carbon for supercapacitors obtained by carbonization of a seaweed biopolymer. Adv. Mater..

[128] Kleszyk P., Ratajczak P., Skowron P., Jagiello J., Abbas Q., Frąckowiak E., Béguin F. (2015). Carbons with narrow pore size distribution prepared by simultaneous carbonization and self-activation of tobacco stems and their application to supercapacitors. Carbon.

[129] Lorenc-Grabowska E., Gryglewicz G., Gryglewicz S. (2004). Development of mesoporosity in activated carbons via coal modification using Ca- and Fe-exchange. Microp. Mesop. Mat..

[130] Gryglewicz G., Lorenc-Grabowska E. (2004). Mesoporous activated carbons from Ca and Fe exchanged sub-bituminous and bituminous coals. Carbon.

[131] Yoshizawa N., Yamada Y., Furuta T., Shiraishi M., Kojima S., Tamai H., Yasuda H. (1997). Coal-based activated carbons prepared with organometallics and their mesoporous structure. Energy Fuels.

[132] Wang L., Sun F., Hao F., Qu Z., Gao J., Liu M., Wang K., Zhao G., Qin Y. (2020). A green trace K_2_CO_3_ induced catalytic activation strategy for developing coal-converted activated carbon as advanced candidate for CO_2_ adsorption and supercapacitors. Chem. Eng. J..

[133] Liu Z., Ling L., Qiao W., Lu C., Wu D., Liu L. (1999). Effects of various metals and their loading methods on the mesopore formation in pitch-based spherical activated carbon. Carbon.

[134] Liu Z., Ling L., Qiao W., Liu L. (1999). Preparation of pitch-based spherical activated carbon with developed mesopore by the aid of ferrocene. Carbon.

[135] Tamai H., Kakii T., Hirota Y., Kumamoto T., Yasuda H. (1996). Synthesis of extremely large mesoporous activated carbon and its unique adsorption for giant molecules. Chem. Mater..

[136] El-Merraoui M., Tamai H., Yasuda H., Kanata T., Mondori J., Nadai K., Kaneko K. (1998). Pore structures of activated carbon fibers from organometallics/pitch composites by nitrogen adsorption. Carbon.

[137] Diez N., Alvarez P., Granda M., Blanco C., Gryglewicz G., Wróbel-Iwaniec I., Śliwak A., Machnikowski J., Menendez R. (2014). Tailoring micro-mesoporosity in activated carbon fibers to enhance SO_2_ catalytic oxidation. J. Colloid Interface Sci..

[138] Basova Y.V., Edie D.D., Badheka P.Y., Bellam H.-C. (2005). The effect of precursor chemistry and preparation conditions on the formation of pore structure in metal-containing carbon fibers. Carbon.

[139] Oya A., Yoshida S., Alcaniz-Monge J., Linares-Solano A. (1995). Formation of mesopores in phenolic resin-derived carbon fiber by catalytic activation using cobalt. Carbon.

[140] Marsh H., Diez M.A., Kuo K., Lahaye J., Ehrburgor P. (1991). Specific Reactivities of Pure Carbon of Diverse Origins. Fundamental Issues in Control of Carbon Gasification Reactivity.

[141] Kaneko K., Ishii C. (1992). Superhigh surface area determination of microporous solids. Colloids Surfaces.

[142] Barbieri O., Hahn M., Herzog A., Kötz R. (2005). Capacitance limits of high surface area activated carbons for double layer capacitors. Carbon.

[143] Salitra G., Soffer A., Eliad L., Cohen Y., Aurbach D. (2000). Carbon electrodes for double-layer capacitors i. relations between ion and pore dimensions. J. Electrochem. Soc..

[144] Eliad L., Salitra G., Soffer A., Aurbach D. (2001). Ion sieving effects in the electrical double layer of porous carbon electrodes: Estimating effective ion size in electrolytic solutions. J. Phys. Chem. B.

[145] Eliad L., Pollak E., Levy N., Salitra G., Soffer A., Aurbach D. (2006). Assessing optimal pore-to-ion size relations in the design of porous poly(vinylidene chloride) carbons for EDL capacitors. Appl. Phys. A.

[146] Thürmer S., Kobayashi Y., Ohba T., Kanoh H. (2015). Pore-size dependent effects on structure and vibrations of 1-ethyl-3-methylimidazolium tetrafluoroborate in nanoporous carbon. Chem. Phys. Lett..

[147] Raymundo-Piňero E., Gao Q., Béguin F. (2013). Carbons for supercapacitors obtained by one-step pressure induced oxidation at low temperature. Carbon.

[148] Ania C.O., Pernak J., Stefaniak F., Raymundo-Piñero E., Béguin F. (2006). Solvent-free ionic liquids as in situ probes for assessing the effect of ion size on the performance of electrical double layer capacitors. Carbon.

[149] Mysyk R., Gao Q., Raymundo-Piñero E., Béguin F. (2012). Microporous carbons finely-tuned by cyclic high-pressure low-temperature oxidation and their use in electrochemical capacitors. Carbon.

[150] Ruiz V., Pandolfo A.G. (2010). Polyfurfuryl alcohol derived activated carbons for high power electrical double layer capacitors. Electrochim. Acta.

[151] Pohlmann S., Lobato B., Centeno T.A., Balducci A. (2013). The influence of pore size and surface area of activated carbons on the performance of ionic liquid based supercapacitors. Phys. Chem. Chem. Phys..

[152] Mousavi M.P.S., Wilson B.E., Kashefolgheta S., Anderson E.L., He S., Bühlmann P., Stein A. (2016). Ionic liquids as electrolytes for electrochemical double-layer capacitors: Structures that optimize specific energy. ACS Appl. Mater. Interfaces.

[153] Decaux C., Matei Ghimbeu C., Dahbi M., Anouti M., Lemordant D., Béguin F., Vix-Guterl C., Raymundo-Piñero E. (2014). Influence of electrolyte ion–solvent interactions on the performances of supercapacitors porous carbon electrodes. J. Power Sources.

[154] Raymundo-Piñero E., Kierzek K., Machnikowski J., Béguin F. (2006). Relationship between the nanoporous texture of activated carbons and their capacitance properties in different electrolytes. Carbon.

[155] Qu D., Shi H. (1998). Studies of activated carbons used in double-layer capacitors. J. Power Source.

[156] Endo M., Maeda T., Takeda T., Kim Y.J., Koshiba K., Hara H., Dresselhaus M.S. (2001). Capacitance and pore-size distribution in aqueous and nonaqueous electrolytes using various activated carbon electrodes. J. Power Source.

[157] Boujibar O., Ghamouss F., Ghosh A., Achak O., Chafik T. (2019). Activated carbon with exceptionally high surface area and tailored nanoporosity obtained from natural anthracite and its use in supercapacitors. J. Power Source.

[158] Kandasamy A., Ramasamy T., Samrin A., Narayanasamy P., Mohan R., Bazaka O., Levchenko I., Bazaka K., Mohandas M. (2020). Hierarchical doped gelatin-derived carbon aerogels: Three levels of porosity for advanced supercapacitors. Nanomaterials.

[159] Hirose E., Nomoto S., Yoshioka K. (2001). Activated Carbon for Use in Electric Double Layer Capacitor and Method of Producing the Same. Patent.

[160] Endo M., Koyama S., Noguchi M., Oki N. (2001). Manufacturing Method of Electrolytic Capacitor, Rectifier, Detector, Switchgear, Light-Sensitive Device or Thermosensitive Device. Patent.

[161] Sonobe N., Nagai A., Aida Tomoyuki T., Noguchi M., Iwaida M., Komazawa E. (2008). Method of Producing A Carbon Material for An Electric double Layer Capacitor Electrode. Patent.

[162] Qu D. (2002). Studies of the activated carbons used in double-layer supercapacitors. J. Power Source.

[163] Torchała K., Kierzek K., Machnikowski J. (2012). Capacitance behavior of KOH activated mesocarbon microbeads in different aqueous electrolytes. Electrochim. Acta.

[164] Kim Y.-J., Horie Y., Matsuzawa Y., Ozaki S., Endo M., Dresselhaus M.S. (2004). Structural features necessary to obtain a high specific capacitance in electric double layer capacitors. Carbon.

[165] Menéndez J.A., Phillips J., Xia B., Radovic L.R. (1996). On the modification and characterization of chemical surface properties of activated carbon: In the search of carbons with stable basic properties. Langmuir.

[166] Menéndez J.A., Xia B., Phillips J., Radovic L.R. (1997). On the modification and characterization of chemical surface properties of activated carbon: Microcalorimetric, electrochemical, and thermal desorption probes. Langmuir.

[167] Tian Y., Song Y., Tang Z., Guo Q., Liu L. (2008). Influence of high temperature treatment of porous carbon on the electrochemical performance in supercapacitor. J. Power Source.

[168] Ruch P.W., Hahn M., Rosciano F., Holzapfel M., Kaiser H., Scheifele W., Schmitt B., Novák P., Kötz R., Wokaun A. (2007). In situ X-ray diffraction of the intercalation of (C_2_H_5_)_4_N^+^ and BF_4_^−^ into graphite from acetonitrile and propylene carbonate based supercapacitor electrolytes. Electrochim. Acta.

[169] Hahn M., Barbieri O., Campana F.P., Kötz R., Gallay R. (2006). Carbon based double layer capacitors with aprotic electrolyte solutions: The possible role of intercalation/insertion processes. Appl. Phys. A.

[170] Iwaida M., Koyama S., Murakami K. (2004). Polarizable Electrode for Electric Double Layer Capacitor and Electric Double Layer Capacitor Using it. Patent.

[171] Iwaida M., Koyama S., Murakami K., Otsuka K., Ozaki K., Tsutsui M. (2004). Activated Carbon, Polarizable Electrode for Electric Double-Layer Capacitor, and Electric Double-Layer Capacitor Using the Same. Patent.

[172] Tanahashi I. (1990). Electrochemical characterization of activated carbon-fiber cloth polarizable electrodes for electric double-layer capacitors. J. Electrochem. Soc..

[173] Ishikawa M., Sakamoto A., Monta M., Matsuda Y., Ishida K. (1996). Effect of treatment of activated carbon fiber cloth electrodes with cold plasma upon performance of electric double-layer capacitors. J. Power Source.

[174] Hsieh C.-T., Teng H. (2002). Influence of oxygen treatment on electric double-layer capacitance of activated carbon fabrics. Carbon.

[175] Babel K., Jurewicz K. (2004). KOH activated carbon fabrics as supercapacitor material. J. Phys. Chem. Solids.

[176] Burke A. (2007). R&D considerations for the performance and application of electrochemical capacitors. Electrochim. Acta.

[177] Pandolfo A.G., Hollenkamp A.F. (2006). Carbon properties and their role in supercapacitors. J. Power Source.

[178] Wang K.-P., Teng H. (2006). The performance of electric double layer capacitors using particulate porous carbons derived from PAN fiber and phenol-formaldehyde resin. Carbon.

[179] Torchała K., Kierzek K., Gryglewicz G., Machnikowski J. (2015). Narrow-porous pitch-based carbon fibers of superior capacitance properties in aqueous electrolytes. Electrochim. Acta.

[180] Bleda-Martínez M.J., Lozano-Castelló D., Cazorla-Amorós D., Morallón E. (2010). Kinetics of double-layer formation: Influence of porous structure and pore size distribution. Energy Fuels.

[181] Endo M., Kim Y.J., Maeda T., Koshiba K., Katayam K., Dresselhaus M.S. (2001). Morphological effect on the electrochemical behavior of electric double-layer capacitors. J. Mater. Res..

[182] Egashira M., Izumi T., Yoshimoto N., Morita M. (2016). Determining water content in activated carbon for double-layer capacitor electrodes. J. Power Source.

[183] Kurzweil P., Chwistek M. (2008). Electrochemical stability of organic electrolytes in supercapacitors: Spectroscopy and gas analysis of decomposition products. J. Power Source.

[184] Hahn M., Würsig A., Gallay R., Novák P., Kötz R. (2005). Gas evolution in activated carbon/propylene carbonate based double-layer capacitors. Electrochem. Commun..

[185] Nguyen Q.D., Wu Y.-H., Wu T.-Y., Deng M.-J., Yang C.-H., Chang J.-K. (2016). Gravimetric/volumetric capacitances, leakage current, and gas evolution of activated carbon supercapacitors. Electrochim. Acta.

[186] Azaïs P., Duclaux L., Florian P., Massiot D., Lillo-Rodenas M.-A., Linares-Solano A., Peres J.-P., Jehoulet C., Béguin F. (2007). Causes of supercapacitors ageing in organic electrolyte. J. Power Source.

[187] Morimoto T., Hiratsuka K., Sanada Y., Kurihara K. (1996). Electric double-layer capacitor using organic electrolyte. J. Power Source.

[188] Jerabek E.C., Mansfield S.F. (2000). Method of Making an Ultracapacitor Electrode. US Patent.

[189] Yoshida A., Tanahashi I., Nishino A. (1990). Effect of concentration of surface acidic functional groups on electric double-layer properties of activated carbon fibers. Carbon.

[190] Xu K., Ding M.S., Richard Jow T. (2001). A better quantification of electrochemical stability limits for electrolytes in double layer capacitors. Electrochim. Acta.

[191] Hahn M., Kötz R., Gallay R., Siggel A. (2006). Pressure evolution in propylene carbonate based electrochemical double layer capacitors. Electrochim. Acta.

[192] Kötz R., Hahn M., Ruch P., Gallay R. (2008). Comparison of pressure evolution in supercapacitor devices using different aprotic solvents. Electrochem. Commun..

[193] Pillay B. (1996). The influence of side reactions on the performance of electrochemical double-layer capacitors. J. Electrochem. Soc..

[194] Nakamura M., Nakanishi M., Yamamoto K. (1996). Influence of physical properties of activated carbons on characteristics of electric double-layer capacitors. J. Power Source.

[195] Lu B., Hu L., Yin H., Mao X., Xiao W., Wang D. (2016). Preparation and application of capacitive carbon from bamboo shells by one step molten carbonates carbonization. Int. J. Hydrog Energy.

[196] Sugo N., Iwasaki H., Uehara G. (2004). Activated Carbon, A Method of Manufacturing the Same, A Polarizable Electrode and An Electric Double Layer Capacitor. Patent.

[197] Sugo N., Iwasaki H., Uehara G. (2006). Activated Carbon, Process for Producing the Same, Polarizable Electrode, and Electric Double Layer Capacitor. Patent.

[198] Qu J., Geng C., Lv S., Shao G., Ma S., Wu M. (2015). Nitrogen, oxygen and phosphorus decorated porous carbons derived from shrimp shells for supercapacitors. Electrochim. Acta.

[199] Huang C., Sun T., Hulicova-Jurcakova D. (2013). Wide electrochemical window of supercapacitors from coffee bean-derived phosphorus-rich carbons. ChemSusChem.

[200] Hulicova-Jurcakova D., Puziy A.M., Poddubnaya O.I., Suárez-García F., Tascón J.M.D., Lu G.Q. (2009). Highly stable performance of supercapacitors from phosphorus-enriched carbons. J. Am. Chem. Soc..

